# Type IX Secretion System Cargo Proteins Are Glycosylated at the C Terminus with a Novel Linking Sugar of the Wbp/Vim Pathway

**DOI:** 10.1128/mBio.01497-20

**Published:** 2020-09-01

**Authors:** Paul D. Veith, Mikio Shoji, Richard A. J. O’Hair, Michael G. Leeming, Shuai Nie, Michelle D. Glew, Gavin E. Reid, Koji Nakayama, Eric C. Reynolds

**Affiliations:** a Oral Health Cooperative Research Centre, Melbourne Dental School, Bio21 Institute, The University of Melbourne, Melbourne, Victoria, Australia; b Department of Microbiology and Oral Infection, Graduate School of Biomedical Sciences, Nagasaki University, Nagasaki, Japan; c School of Chemistry, Bio21 Molecular Science and Biotechnology Institute, University of Melbourne, Melbourne, Victoria, Australia; d Melbourne Mass Spectrometry and Proteomics Facility, Bio21 Molecular Science and Biotechnology Institute, University of Melbourne, Melbourne, Victoria, Australia; e Department of Biochemistry and Molecular Biology, University of Melbourne, Melbourne, Victoria, Australia; University of Georgia

**Keywords:** *Porphyromonas gingivalis*, *Tannerella forsythia*, glycoprotein, type IX secretion system, lipopolysaccharide

## Abstract

Porphyromonas gingivalis and Tannerella forsythia use the type IX secretion system to secrete cargo proteins to the cell surface where they are anchored via glycolipids. In P. gingivalis, the glycolipid is anionic lipopolysaccharide (A-LPS), of partially known structure. Modified cargo proteins were deglycosylated using trifluoromethanesulfonic acid and digested with trypsin or proteinase K. The residual modifications were then extensively analyzed by tandem mass spectrometry. The C terminus of each cargo protein was amide-bonded to a linking sugar whose structure was deduced to be 2-*N*-seryl, 3-*N*-acetylglucuronamide in P. gingivalis and 2-*N*-glycyl, 3-*N*-acetylmannuronic acid in T. forsythia. The structures indicated the involvement of the Wbp pathway to produce 2,3-di-*N*-acetylglucuronic acid and a WbpS amidotransferase to produce the uronamide form of this sugar in P. gingivalis. The *wbpS* gene was identified as PGN_1234 as its deletion resulted in the inability to produce the uronamide. In addition, the P. gingivalis *vimA* mutant which lacks A-LPS was successfully complemented by the T. forsythia *vimA* gene; however, the linking sugar was altered to include glycine rather than serine. After removal of the acetyl group at C-2 by the putative deacetylase, VimE, VimA presumably transfers the amino acid to complete the biosynthesis. The data explain all the enzyme activities required for the biosynthesis of the linking sugar accounting for six A-LPS-specific genes. The linking sugar is therefore the key compound that enables the attachment of cargo proteins in P. gingivalis and T. forsythia. We propose to designate this novel linking sugar biosynthetic pathway the Wbp/Vim pathway.

## Abstract (precis)

Porphyromonas gingivalis and Tannerella forsythia, two pathogens associated with severe gum disease, use the type IX secretion system (T9SS) to secrete and attach toxic arrays of virulence factor proteins to their cell surfaces. The proteins are tethered to the outer membrane via glycolipid anchors that have remained unidentified for more than 2 decades. In this study, the first sugar molecules (linking sugars) in these anchors are identified and found to be novel compounds. The novel biosynthetic pathway of these linking sugars is also elucidated. A diverse range of bacteria that do not have the T9SS were found to have the genes for this pathway, suggesting that they may synthesize similar linking sugars for utilization in different systems. Since the cell surface attachment of virulence factors is essential for virulence, these findings reveal new targets for the development of novel therapies.

## INTRODUCTION

Porphyromonas gingivalis and Tannerella forsythia are Gram-negative, anaerobic oral bacteria strongly associated with periodontitis in humans (1, 2). Both microbes possess a type IX secretion system (T9SS) which they use to secrete and attach virulence-associated cargo proteins to their cell surfaces via a glycolipid, which in P. gingivalis is anionic lipopolysaccharide (A-LPS) (3–5). Periodontitis associated with these microbes has been linked to an increased risk of cardiovascular diseases, certain cancers, preterm birth, rheumatoid arthritis, and dementia (6–9).

P. gingivalis LPS has been detected in two forms, the first, O-LPS is composed of lipid A, core oligosaccharide, and an O antigen that consists of a repeating tetrasaccharide of GalNAc, Rha, Glc, and Gal (10, 11). Originally A-LPS was reported to have the same lipid A and core as O-LPS but a different polysaccharide composed of a repeating branched phosphomannan (12, 13). More recently, A-LPS has been suggested to contain a repeating tetrasaccharide similar in structure to that of the O antigen (14). At a practical level, A-LPS is defined as the form of LPS that is recognized by the monoclonal antibody (MAb) 1B5 (15, 16). The use of this antibody has linked A-LPS to the modification used to anchor T9SS cargo proteins to the cell surface and enabled the identification of genes required for A-LPS biosynthesis (14, 17). Genes specific to A-LPS biosynthesis are implicated in the synthesis of the repeating polysaccharide portion of A-LPS since the remaining parts of the structure (core and lipid A) are in common with O-LPS. The known A-LPS-specific genes encode the predicted glycosyltransferases WbaP, GtfC, GtfF, and VimF (14), components of the Wbp pathway WbpA, WbpB, WbpD, and WbpE (PorR) (18), and, additionally, two proteins of unknown function, VimA and VimE (19). VimA and VimE are reported to be a putative acetyl-coenzyme A (CoA) transferase and a carbohydrate esterase, respectively (20). The *vimA* mutant is linked to a wide range of phenotypes; however, its specific role in A-LPS biosynthesis has not been elucidated (20, 21). The product of the Wbp pathway is known to be a di-*N*-acetylated uronic acid component of O antigen in other organisms (22, 23), and therefore a related sugar is hypothesized to be a component of A-LPS polysaccharide in P. gingivalis (18). Together, the presence of these A-LPS specific glycosyltransferases and biosynthetic enzymes indicate that there is more to the A-LPS polysaccharide structure than the originally reported phosphomannan.

The T9SS is found in many diverse Gram-negative bacteria of the *Bacteroidetes* phylum and is typically involved in the secretion of between 10 and over 100 different substrates (cargo proteins) per species (24, 25). The function of the T9SS is dependent on the expression of PorE (PG1058), PorF (PG0534), PorG (PG0189, PGN_0297), PorK, PorL, PorM, PorN, PorP, PorQ, PorT, PorU, PorV, PorW, PorZ, and Sov (3, 26–28). In P. gingivalis mutants lacking any one of these components, cargo proteins, such as the gingipains, are trapped inside the periplasm in an immature nonglycosylated form, and the electron-dense surface layer (EDSL) comprising the mature, A-LPS-modified cargo proteins is not produced (29). These mutants, along with A-LPS mutants, are unable to produce black pigmented colonies on blood agar since surface-attached gingipains are required to accumulate the heme pigment on the cell surface (17). In T. forsythia, T9SS mutants lacking orthologs of PorK, PorT, PorU, and Sov also do not produce a surface layer, confirming that the known constituents of the surface layer (TfsA and TfsB) are T9SS cargo proteins (30, 31).

The T9SS cargo proteins have a C-terminal signal of approximately 80 amino acids that directs secretion through the outer membrane (OM) pore which is proposed to be the Sov (SprA) protein (32). SprA was isolated as two distinct complexes, one with PorV and the other with a novel component of the T9SS, referred to as the plug. The cryo-electron microscopy (cryo-EM) structure of these two complexes indicated that SprA forms a 36-stranded OM β-barrel with a large internal pore. These structures demonstrate a pathway for substrates to cross the OM and bind to PorV (32). PorV has previously been shown to bind to substrates on the cell surface and was proposed to shuttle substrates from the pore to the attachment complex comprising PorU, PorZ, PorQ, and additional PorV (33). PorU is a member of the gingipain family of cysteine proteinases which have recently been shown to be transpeptidases (34). Consistent with this, PorU has been implicated as a novel Gram-negative sortase that cleaves the T9SS signal at a moderately conserved site and conjugates the new C terminus to A-LPS (35, 36). Mutant strains lacking genes that are essential for either A-LPS biosynthesis or the T9SS are nonpigmented. In P. gingivalis, mass spectrometry (MS) analyses of the A-LPS-modified proteins after deglycosylation with trifluoromethanesulfonic acid (TFMS) demonstrated that the mature C terminus of each protein was linked to an A-LPS fragment of 648 Da comprised of three units with masses of 104 Da, 198 Da, and 346 Da (36). The 104-Da unit was shown to be linked to the protein via a peptide bond.

In this study, we conducted extensive MS analyses of these LPS fragments isolated from both P. gingivalis and T. forsythia and report the putative structures of the linking sugars and their biosynthesis via the novel Wbp/Vim pathway.

## RESULTS

**FIG 1:**
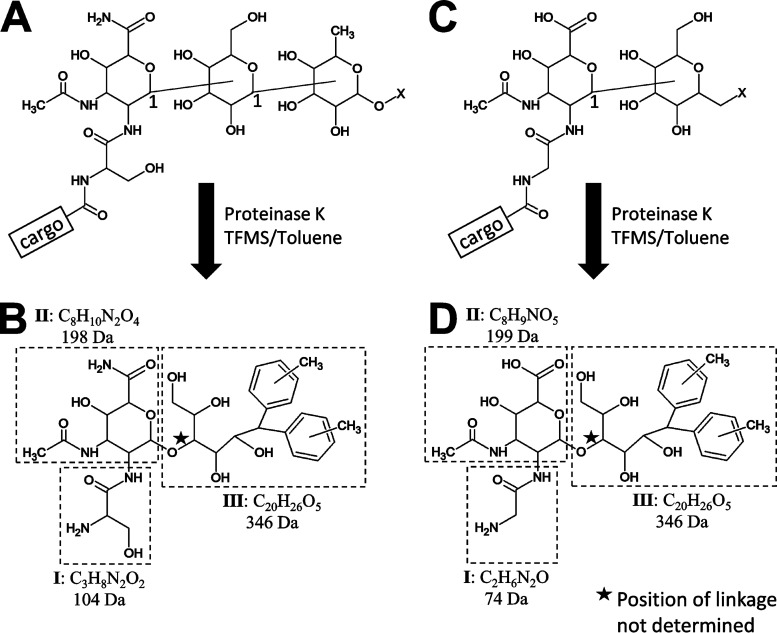
Proposed overall structure of LPS fragments. Proposed structures of LPS fragments from P. gingivalis (A) and T. forsythia (C) in their native contexts bonded to a cargo protein. The major reaction products after cleavage with proteinase K and deglycosylation with TFMS in the presence of toluene are shown for P. gingivalis (B) and T. forsythia (D). The position of the glycosidic bonds (bonds between sugars) are unknown except for the C-1 positions as shown (A and C). The glycosidic bond position shown in the reaction product (B and D), indicated with a star, is only one of several possibilities. The three components, I, II, and III, are indicated by dashed boxes and represent the major CID fragments observed. For accurate mass data for the determination of molecular formulae, see the main text and Tables 2 and 3.

In this study, we present detailed MS analyses of the LPS fragments isolated from modified cargo proteins of P. gingivalis and T. forsythia. The proposed structures of these fragments combined with the structure of their major TFMS-cleaved products are shown in Fig. 1. The elucidation of these structures is described below.

### Determining the accurate mass and molecular formula.

Previously, P. gingivalis T9SS cargo proteins were found to be modified at their matured C termini with A-LPS. A fragment of A-LPS that remained bonded to these proteins after deglycosylation with TFMS was shown to be composed of three components, I, II, and III, with masses of 104, 198, and 346 Da, respectively, with component I suggested to be a serinamide that was amide linked to the new C terminus of the cargo proteins (36). In this study, the A-LPS fragment was released by proteinase K cleavage; detailed MS^n^ analysis was conducted using a linear ion trap quadrupole Fourier-transform ion cyclotron resonance (LTQ-FTICR) mass spectrometer (see analysis of components I and II below), and high-resolution spectra were acquired for the determination of accurate mass. The A-LPS fragment exhibited an *m/z* of 649.3070 (*z *= 1+) and collision-induced dissociation (CID) fragmentation of this peak produced a major peak at *m/z* 303.1298, which matched best to the formula C_11_H_19_N_4_O_6_ with an error of 0.37 ppm. Taking this into consideration, the best match to the precursor ion (649 Da) was C_31_H_45_N_4_O_11_ with an error of 1.4 ppm. The difference between these molecular formulae is C_20_H_26_O_5_, (346 Da) corresponding to component III (Fig. 1B). MS^3^ of the *m/z* 303 ion produced a major peak at *m/z* 199.0713, corresponding to component II which, after the proton was subtracted, uniquely matched to C_8_H_10_N_2_O_4_, with an error of 0.17 ppm (Fig. 1B). Subtracting this from the formula of the *m/z* 303 ion gives the molecular formula of component I, C_3_H_8_N_2_O_2_ (Fig. 1B). Interestingly, MS^3^ of the dehydration product (649 →631) exhibited a peak at *m/z* 209.1325, which uniquely matched to C_16_H_17_ with an error of 0.11 ppm. Within the scope of the whole study, this peak was observed only when ions that included component III were fragmented, indicating that the C_16_H_17_ fragment derived from component III.

### TFMS deglycosylation in the presence of ethylbenzene reveals the underlying chemistry.

**FIG 2:**
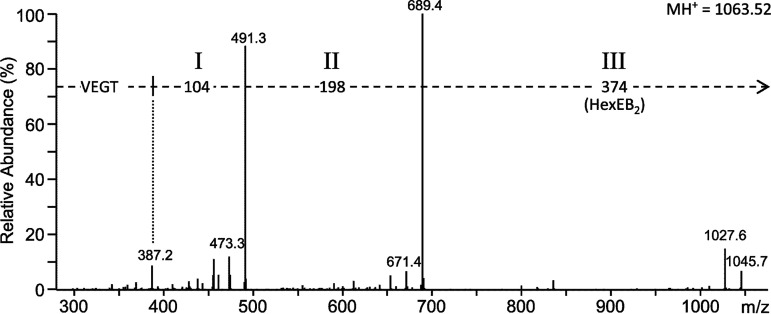
Deglycosylation in the presence of ethylbenzene demonstrates aryl adducts. Orbitrap LC-MS/MS analysis of a modified RgpB C-terminal peptide with the sequence VEGT after deglycosylation in the presence of ethylbenzene (EB) instead of toluene. The mass of component III was increased by 28 Da (compare with value shown in Fig. 1B), suggesting the presence of two EB molecules and a hexose residue. The arrow indicates that the mass difference of 374 Da is relative to the precursor *m/z* of 1,063.52.

The identification of the C_16_H_17_ fragment was interesting as it suggested that this group may be a novel hydrophobic anchor that is inserted into the OM. An alternative explanation, however, was the possibility that this group was derived from the toluene (C_7_H_8_) used as a free radical scavenger in the deglycosylation reaction. To test this, new P. gingivalis samples were prepared with the deglycosylation step being conducted in the presence of ethylbenzene (C_8_H_10_), abbreviated below as EB, rather than toluene (Tol). Liquid chromatography tandem MS (LC-MS/MS) analyses of the trypsin-digested samples failed to detect any of the previously identified peptides with the mass difference of 630 Da for the modification. Instead, modified C-terminal peptides were found to have an increased mass difference of 658 Da, indicating that the presence of scavenger (toluene or EB) was causing an artefactual modification. The mass difference between toluene and EB is 14 Da; however, the observed mass shift was 28 Da, suggesting that the artifact included two molecules of toluene or EB. As expected, precursor ions would lose 374 Da rather than 346 Da, demonstrating that the artifact was part of component III (Fig. 2). Subtracting Tol_2_ (184 Da) or EB_2_ (212 Da) from the component III masses of 346 Da and 374 Da, respectively, gives C_6_H_10_O_5_ (162 Da), which likely corresponds to a hexose (Hex) residue (Fig. 1B and 2).

**FIG 3:**
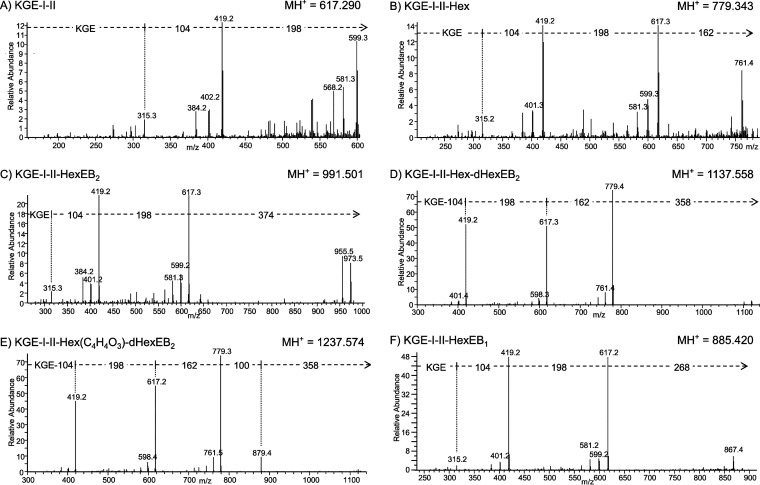
(A to F) MS/MS spectra of the P27 C-terminal peptide with various modifications. A Q-Sepharose fraction that included P27 (PG1795) was deglycosylated in the presence of ethylbenzene for ∼25 min. The deglycosylated sample was fractionated by SDS-PAGE, and the band containing P27 was digested with trypsin to produce modified C-terminal peptides with the sequence KGE and analyzed by LC-MS/MS (CID) on an Orbitrap instrument. The assignment of each sequence is provided in the top left of each spectrum. The precursor ion peaks are not visible due to their fragmentation, but their approximate locations are at the ends of the arrows. The mass of the protonated precursor ion (MH^+^) is provided in the top right of each spectrum. The mass of each component is as follows: component I, 104 Da; component II, 198 Da; Hex, 162 Da; HexEB_2_, 374 Da; dHexEB_2_, 358 Da; C_4_H_4_O_3_, 100 Da; HexEB_1_, 268 Da.

To identify further potential sugars, TFMS deglycosylation of P. gingivalis proteins purified from outer membrane vesicles (OMVs) was conducted in a time series in the presence of EB. Trypsin-digested samples were again analyzed by LC-MS/MS (Orbitrap) analyses. The C-terminal peptides of RgpB, P27, and PG0553 produced the most comprehensive data. The RgpB C-terminal peptide (VEGT) was analyzed first. The first time point of ∼25 min provided the most intense peaks for all the modified peptides that were identified, as well as proportionally higher intensities for the larger species, with the peaks at *m/z* 1,209 and *m/z* 1,309 exhibiting an intensity of 4.6% and 1.9% relative to the most abundant form at *m/z* 1,063 (Table 1). Presumably, the longer cleavage times caused a greater degree of cleavage as well as a greater diversity of unwanted reactions. Therefore, an ∼25-min reaction time was chosen for all subsequent experiments. The MS/MS data for the *m/z* 1,209 peak indicated the presence of a deoxyhexose (dHex) while, in addition, the *m/z* 1,309 peak also contained C_4_H_4_O_3_ (Table 1). The same results were found for the C-terminal peptides of P27 and PG0553, and the MS/MS spectra for P27 are shown in Fig. 3. The MS/MS spectrum of the most intense peak of *m/z* 991 corresponds to the P27 C-terminal peptide KGE linked to components I, II, and III (KGE-I-II-HexEB_2_) (Fig. 3C). A weaker peak at *m/z* 617 was found to correspond to just KGE-I-II (Fig. 3A). Since this peptide was observed at the same retention time but was missing the hydrophobic HexEB_2_ it was concluded to be an in-source decay fragment. Helpfully, the *m/z* 779 form consisting of components I, II, and the hexose residue (162 Da) without the EB_2_ artifact was also observed (Fig. 3B). This peak was also concluded to be an in-source decay fragment as it had low intensity and the same retention time as the compound shown in Fig. 3D. This peak at *m/z* 1,137 was of higher mass than the major form by 146.057 Da, which accurately matches the mass of a deoxyhexose residue. MS/MS of this form suggested that a deoxyhexose is the next sugar in the chain (Fig. 3D). An additional form at *m/z* 1,237 was 100.016 Da higher, matching uniquely to C_4_H_4_O_3_, which may represent a residue of an organic acid such as succinate. MS/MS of this form first lost 358 Da (dHexEB_2_) and then 100 Da, suggesting that the C_4_H_4_O_3_ group was bonded to the hexose residue (Fig. 3E). Consistent with this, an MS peak at *m/z* 1,091 corresponding to KGE-I-II-Hex(C_4_H_4_O_3_)EB_2_ was also observed (spectrum not shown). In this case, the MS/MS data did not support a separate loss of 100 Da; rather, the 100 Da was lost with the HexEB_2_. Analogous results were found for the C-terminal peptides of all three proteins (data not shown), confirming that the A-LPS fragment includes components I, II, hexose, deoxyhexose, and C_4_H_4_O_3._ Despite extensive manual analysis of the data set, further sugars could not be reliably assigned.

**TABLE 1:** TFMS deglycosylation time series for sample containing modified P. gingivalis RgpB

MH^+^ (*m*/*z*)	Assignment^*a*^	Intensity at the indicated reaction time (%)^*b*^			
		25 min	1 h	3 h	16 h
1,063.523	VEGT-I-II-Hex-EB_2_	1.2 × 10^8^ (100)	6.8 × 10^7^ (100)	9.8 × 10^7^ (100)	3.7 × 10^7^ (100)
1,209.582	VEGT-I-II-Hex-dHex-EB_2_	5.4 × 10^6^ (4.6)	3.0 × 10^6^ (4.4)	2.9 × 10^6^ (3.0)	1.2 × 10^6^ (3.2)
1,309.597	VEGT-I-II-Hex(C_4_H_4_O_3_)-dHex-EB_2_	2.2 × 10^6^ (1.9)	5.2 × 10^5^ (0.8)	2.0 × 10^5^ (0.2)	6.5 × 10^4^ (0.2)

a Assignment was accomplished by matching of MS/MS fragments. Components I and II are defined in Fig. 1. Hex, hexose; dHex, deoxyhexose; EB, ethylbenzene.

b The samples were digested with trypsin, and the data for the modified C-terminal peptide VEGT are shown. Values in parentheses are relative to the value for the most abundant form, *m/z* 1,063.

### Modification of *T. forsythia* T9SS substrates.

**FIG 4:**
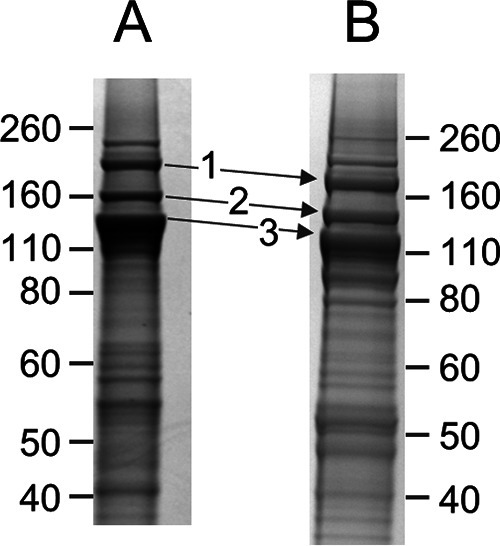
SDS-PAGE of T. forsythia OMVs before and after deglycosylation with TFMS. Samples were electrophoresed and stained with Coomassie blue, and the indicated bands were analyzed by MS. (A) Prior to deglycosylation with TFMS. (B) After deglycosylation with TFMS on a separate gel. The major proteins identified in each band together with their theoretical masses after deglycosylation are the following: band 1, Tanf_02425 (189 kDa); band 2, TfsB (142 kDa); band 3, TfsA (125 kDa).

Since T. forsythia is closely related to *Porphyromonas* species, we next determined whether a similar modification might occur in the cargo proteins of this species. Since the OMVs of this organism are enriched with these cargo proteins (37), we used OMVs as the starting material. Deglycosylation of OMVs with TFMS resulted in a reduction in the molecular weight (MW) of the major high-MW bands, known to correspond to TfsA, TfsB, and Tanf_02425 (37), to values more consistent with their calculated MWs, suggesting that the deglycosylation was successful (Fig. 4). These deglycosylated bands were then digested with trypsin and analyzed by LC-MS/MS.

**FIG 5:**
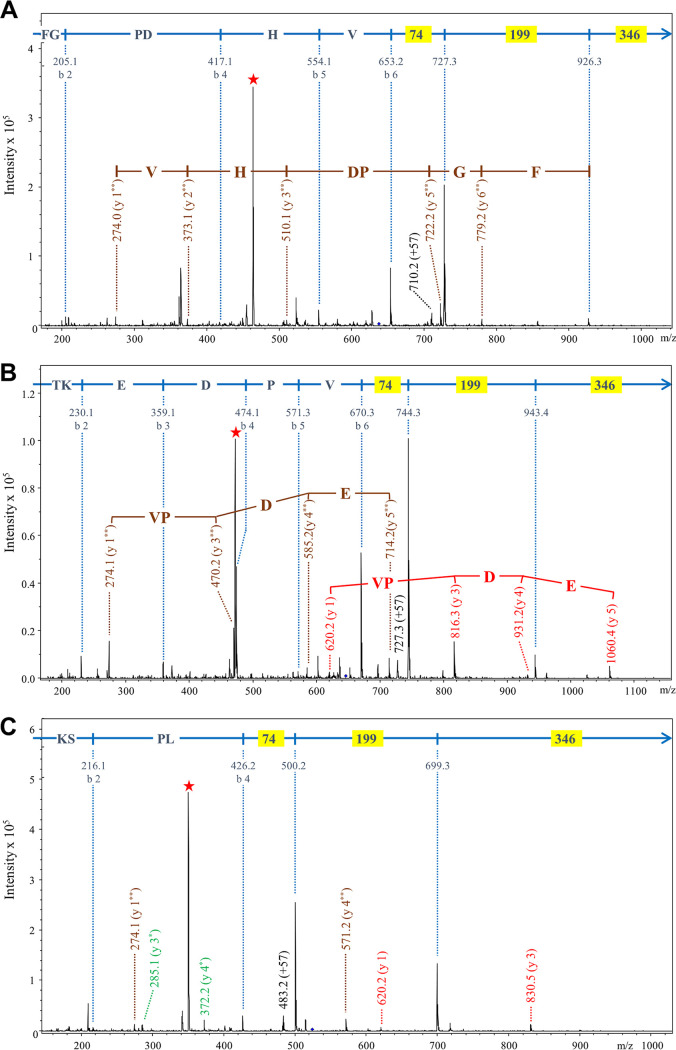
MS/MS analyses of modified C-terminal peptides of T. forsythia cargo proteins. Deglycosylated protein bands were digested with trypsin and analyzed by LC-MS/MS using the HCT Ultra ion trap. Each panel shows the MS/MS spectrum obtained for a modified C-terminal tryptic peptide with a delta mass of +601.2 Da. The protein names and peptide sequences are as follows: Tanf_02425, FGPDHV (A); TfsB, TKEDPV (B); TfsA, KSPL (C). The b-ions and their corresponding sequence assignments are shown in blue, with masses (74, 199, and 346) corresponding to components of the LPS fragment highlighted yellow. The y-ions are labeled such that the ion corresponding to the modification alone is y-1. The y-ions beginning with the intact (619 Da) modification are in red, and are labeled y 1, y 2, etc. The y-ions beginning with the 273-Da component are in brown and are labeled y 1**, y 2**, etc. The y-ions beginning with the 74-Da component are in green and are labeled y 1*, y 2*, etc. The intense peaks labeled with a red star are doubly charged ions that have lost the 346-Da component. The precursor ion peaks are not visible due to their fragmentation, but their approximate locations are at the end of the arrows.

Initially, the data were searched by Mascot using the same delta mass of +630.3 Da used for P. gingivalis; however, no C-terminal peptides were positively identified, suggesting that the modification might be different. We therefore plotted neutral-loss chromatograms of −346 Da for each band corresponding to the loss of component III (data not shown). The neutral-loss chromatogram for band 1 exhibited a strong peak at 33.4 min corresponding to the fragmentation of a compound of *m/z* 637.2. Detailed inspection of the MS/MS spectrum of this compound resulted in the identification of the expected C-terminal peptide of Tanf_02425 with the sequence FGPDHV and a modification delta mass of +601.2 Da, 29 Da less than the P. gingivalis modification. As an addendum to the b-ion series, major ions were observed at further mass differences of 74 and 199 Da (Fig. 5A). The same approach was employed for bands 2 and 3 (Fig. 4B), resulting in the identification of the modified C-terminal peptides of TfsB and TfsA, respectively (Fig. 5B and C). When the data were automatically searched by Mascot using +601.2 Da as an optional C-terminal modification, modified C-terminal peptides were identified for an additional two cargo proteins, Tanf_11855 and Tanf_06020 (37; also data not shown). In each case, the same pattern of peaks was observed, suggesting that the residual modification for these T. forsythia cargo proteins is composed of three components, I, II, and III, with masses of 74 Da, 199 Da and 346 Da, respectively (Fig. 1D).

With P. gingivalis modified C-terminal peptides, the modification could be released by proteinase K cleavage, indicating a peptide bond between the protein C terminus and the 104-Da component of the modification (36). Therefore, we also treated the deglycosylated T. forsythia proteins with proteinase K and analyzed the digestion products with LC-MS/MS. Through searching for the characteristic neutral loss of 346 Da, we observed a singly charged peak at *m/z* 620.3 that produced major product ions at *m/z* 274 (−346 Da) and *m/z* 200 (component II, MH^+^), confirming its identity as the residual modification, which, as in P. gingivalis, appears to be connected to the cargo protein via a peptide (amide) bond (data not shown).

Analyses of the T. forsythia C-terminal peptides by orbitrap LC-MS/MS was conducted using higher resolution (240,000) in the MS/MS scans to allow assignment of molecular formulae. For these spectra, inspection of the peptide a-ions and b-ions across the relevant mass range gave a maximum error of <1 ppm, and therefore these data were searched with a tolerance of 1 ppm. Component I returned C_2_H_6_N_2_O as the only match; component II returned C_8_H_9_NO_5_ as the only match, and component III returned C_20_H_26_O_5_ as the simplest of four conceivable matches (Fig. 1D and Table 2), consistent with the hexose-toluene adduct identified for P. gingivalis.

**TABLE 2:** Accurate mass data from Orbitrap MS/MS of modified T. forsythia peptides

Parameter^*a*^	Value for the component^*b*^			Δ Mass^*c*^
	I (74 Da)	II (199 Da)	III (346 Da)	
Mass (Da)				
TfsA	74.04808	199.04813	346.17800	601.26342
TfsB	74.04797	199.04816	346.17792	601.26367
Tanf_02425	74.04807	199.04811	346.17832	601.26385
Avg	74.04804	199.04813	346.17808	601.26365
Molecular formula	C_2_H_6_N_2_O	C_8_H_9_NO_5_	C_20_H_26_O_5_^*d*^	C_30_H_39_O_10_^*e*^
DBE	1	5	8	13
Error (ppm)	−0.4	−0.3	−0.2	−0.2

a The molecular formulae, double-bond equivalents (DBE), and associated errors were calculated using a molecular weight-to-formula tool (Bruker). The settings allowed for unlimited C, H, N, and O and a maximum of P_3_, S_3_, and F_3_. Fluorine was included due to the utilization of TFMS in the deglycosylation reactions. Phosphorus, if present, was assumed to be associated with at least 3 oxygens (most likely as phosphate). The data were searched at 1 ppm.

b T. forsythia data were obtained from Orbitrap MS^2^ data of the modified C-terminal peptides at a resolution of 240,000. The accurate mass of component I was calculated from the *m/z* value at 274 minus the *m/z* value at 200. The accurate mass of component II was calculated from the *m/z* value at 200 minus H^+^. The accurate mass of component III was taken as the neutral loss of 346 Da.

c The delta mass (Δ mass) for the entire modification was calculated by subtracting the calculated *m/z* of the peptide from the observed *m/z* of the modified peptide and multiplying by z.

d Other possible but unlikely formulae were C_11_H_28_FN_4_O_5_P, C_18_H_28_F_2_O_2_S, and C_14_H_19_FN_10_.

e Other formulae were possible within these error limits.

### Component II.

**FIG 6:**
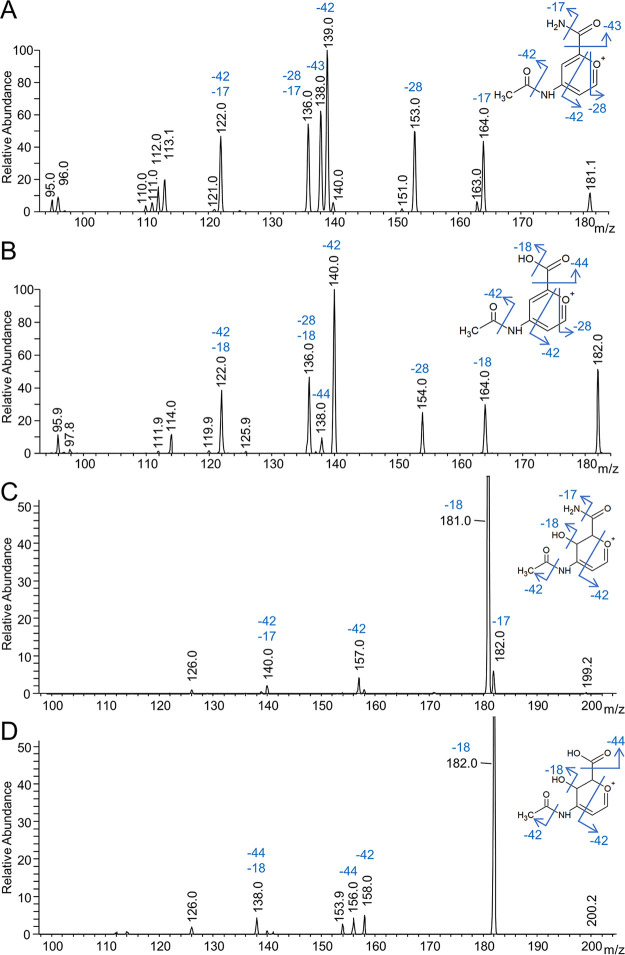
MS^4^ spectra to determine the component II structures. LPS fragments were purified from modified cargo proteins that were deglycosylated and treated with proteinase K. The proposed structure and fragmentation of the precursor ions are shown. All spectra were acquired by direct infusion into the FTICR instrument. The MS^4^ settings were as follows: P. gingivalis, 649.33 *m*/*z* at CID 20.00, 303.00 *m*/*z* at CID 30.00, and 181.00 *m*/*z* at CID 40.00 (A); T. forsythia, 620.50 *m*/*z* at CID 30.00, 274.00 *m*/*z* at CID 30.00, and 182.00 *m*/*z* at CID 30.00 (B); P. gingivalis, 649.33 *m*/*z* at CID 20.00, 303.00 *m*/*z* at CID 30.00, and 199.00 *m*/*z* at CID 30.00 (C); T. forsythia, 620.50 *m*/*z* at CID 30.00, 274.00 *m*/*z* at CID 30.00, and 200.00 *m*/*z* at CID 30.00 (D). These MS^4^ spectra follow the MS^3^ spectra shown in Fig. 7.

The major data collected for components I and II were the detailed MS^n^ analyses of the purified LPS fragments from both species. The tentative structures for component II were *N*-acetyl glucuronamide and *N*-acetyl glucuronic acid for P. gingivalis and T. forsythia, respectively. This was deduced from a large number of MS spectra, mostly at the MS^4^ level. These structures are supported by the following data. The MS^4^ data of dehydrated component II at *m/z* 181 (P. gingivalis) and *m/z* 182 (T. forsythia) proved particularly insightful (Fig. 6A and B), and these data were compared to the fragmentation patterns obtained for synthetic dehydrated glucuronamide and *N*-acetylglucosamine (NAG) (see Fig. S1 in the supplemental material). The precursor ions shown in Fig. 6A and B were assigned to disubstituted pyrylium ions and were found to lose major modules of NH_3_ (−17 Da), CO (−28 Da), C_2_H_2_O (−42 Da), and CHNO (−43 Da) for P. gingivalis and H_2_O (−18 Da), CO (−28 Da), C_2_H_2_O (−42 Da), and CO_2_ (−44 Da) for T. forsythia. The loss of 59 Da shown in Fig. 6A was deemed to correspond to consecutive losses of 42 Da and 17 Da rather than to the loss of acetamide (59 Da). This was supported by MS^n^ analyses of glucuronamide and NAG as the loss of NH_3_ was observed only for glucuronamide, the loss of ketene (−42 Da) was observed only for NAG, and the loss of 59 Da was not observed in either (Fig. S1). This interpretation is also consistent with data shown in Fig. 6B where, instead of a loss of 59 Da, a loss of 60 Da was observed corresponding to consecutive losses of 42 Da and 18 Da.

10.1128/mBio.01497-20.1 FIG S1 MS^n^ of N-acetyl glucosamine and glucuronamide. Download FIG S1, PDF file, 0.2 MB. Copyright © 2020 Veith et al. 2020 Veith et al. This content is distributed under the terms of the Creative Commons Attribution 4.0 International license.

**FIG 7:**
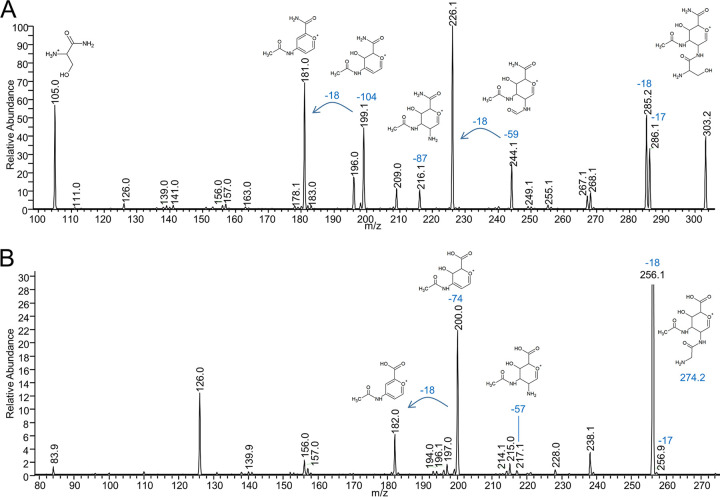
MS^3^ spectra to determine the component I structures. (A) MS^3^ spectrum of *m/z* 303 derived from an A-LPS fragment purified from P. gingivalis (649.33 *m*/*z* at CID 20.00 and 303.00 *m*/*z* at CID 20.00). The proposed structures of the precursor ion (*m/z* 303) and fragments of interest are shown. (B) MS^3^ spectrum of *m/z* 274 derived from an LPS fragment purified from T. forsythia (620.50 *m*/*z* at CID 30.00 and 274.00 *m*/*z* at CID 30.00). The proposed structures of the precursor ion (*m/z* 274) and fragments of interest are shown. Both spectra were acquired by direct infusion into the FTICR instrument.

The peak at *m/z* 138 in both spectra (Fig. 6A and B) is proposed to be an *N*-acetyl pyrylium ion resulting from the loss of cyanic acid (−43 Da) in P. gingivalis or the loss of CO_2_ (−44 Da) in T. forsythia. The favorable formation of this *m/z* 138 ion was demonstrated by the loss of formaldehyde (−30 Da) in the fragmentation of dehydrated NAG (Fig. S1B), while the loss of cyanic acid (−43 Da) could be replicated from dehydrated glucuronamide (Fig. S1D). The loss of CO (−28 Da) observed in both P. gingivalis and T. forsythia (Fig. 6A and B) was deduced to be from the ring and was supported by the same loss from both NAG and glucuronamide (Fig. S1). The suspected loss of CO from the ring infers a nonsubstituted carbon adjacent to the ring oxygen, presumably C-1. The loss of ketene (−42 Da) was the most favorable loss in both spectra (Fig. 6A and B) and theoretically could be from either the *N*-acetyl group or from ring cleavage. It was concluded that most of the loss was from ring cleavage because the loss of ketene was much less for NAG (Fig. S1B), and ketene was not lost when serinamide or glycinamide was present in the structure (Fig. 7). MS^4^ analyses of the nondehydrated component II ions were dominated by the loss of water (Fig. 6C and D). The next major loss was again ketene (−42 Da), supporting the position of the hydroxyl group at C-4 rather than at C-1.

### Component I.

For both P. gingivalis and T. forsythia, the molecular formula of the first component was unequivocal. Since component I is linked directly to the protein C terminus via an amide (peptide) bond, it is deduced to have a free amine. Furthermore, while major fragment ions were observed for C-terminal peptide +104 Da (P. gingivalis) or +74 Da (T. forsythia), ions were also observed for C-terminal peptide +87 Da (P. gingivalis) or +57 Da (T. forsythia), which matches to serine or glycine, respectively (36), (Fig. 5). The additional 17 Da is inferred to correspond to an additional amine that connects components I and II. For the 74.0480-Da entity, the only metabolite in the Metlin database having two free amines is 2-aminoacetamide (glycinamide). Therefore, since the component I compounds from P. gingivalis and T. forsythia are likely related, we hypothesized that they are glycinamide (T. forsythia) and serinamide (P. gingivalis), respectively.

**FIG 8:**
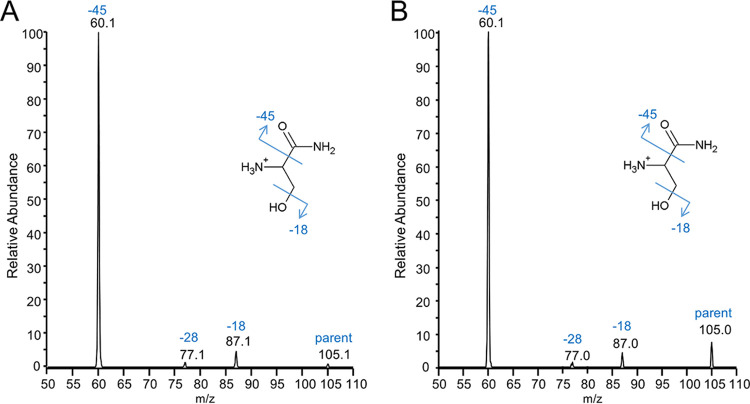
MS/MS analysis of P. gingivalis component I compared to serinamide. (A) MS^2^ spectrum of synthetic serinamide. (B) MS^4^ spectrum of *m/z* 105 derived from the A-LPS fragment purified from P. gingivalis (649.33 *m*/*z* at CID 20.00, 303.00 *m*/*z* at CID 30.00, and 105.00 *m*/*z* at CID 40.00). The proposed structure and fragmentation of the precursor ions (*m/z* 105) are shown. Both spectra were acquired by direct infusion into the FTICR instrument. The MS^4^ spectrum follows the MS^3^ spectrum shown in Fig. 7A.

To confirm the assignment of serinamide, the MS^4^ data of the component I ion (*m/z* 105) from the purified P. gingivalis LPS fragment was analyzed and compared to the MS^2^ spectrum of synthetic serinamide (Fig. 8). The spectra produced indistinguishable profiles, with observed fragments at *m/z* 60 corresponding to the loss of formamide and at *m/z* 87 corresponding to the loss of H_2_O. An unexpected ion at *m/z* 77 was present in both spectra, presumably representing the loss of CO concomitant with molecular rearrangement.

Further confirmation was obtained from the MS^3^ spectra of the linking sugars comprising components I and II of *m/z* 303 (P. gingivalis) and *m/z* 274 (T. forsythia) (Fig. 7). For P. gingivalis, major losses were observed for modules of H_2_O (−18 Da), NH_3_ (−17 Da), C_2_H_5_NO (−59 Da), component II (−198 Da) and putatively serine (−87 Da), and serinamide (−104 Da). The accurate masses of these fragment ions analyzed by Orbitrap are shown in Table 3. The loss of 59 Da was assigned to the serine component of the P. gingivalis modification (Fig. 7A). This preferred cleavage between the carbonyl and alpha carbons is the same as that of serinamide (Fig. 8). The loss of NH_3_ to give a peak at *m/z* 286 could be from either component I or II. MS^4^ of the *m/z* 286 ion gave rise to major losses of H_2_O, further NH_3_, −87 Da, and new major losses at −30 Da and −47 Da while the losses of −59 Da and −104 Da were minimal (Fig. S2). The data are most consistent with the *m/z* 286 ion mostly lacking NH_3_ from serine, with a smaller proportion of molecules having lost NH_3_ from component II. With NH_3_ removed, the losses of 30 Da, 42 Da, and 87 Da are readily matched to cleavages involving the residual serinamide component. The large loss of H_2_O from the *m/z* 286 ion may also be at least partly from the serine side chain. The same serinamide fragmentation was observed in the MS^4^ spectra of the dehydrated ions of *m/z* 285 and *m/z* 267 (Fig. S2). These losses (besides H_2_O) were not observed in the equivalent spectra for T. forsythia (Fig. 7B), further supporting their assignment to the serine moiety in P. gingivalis. For T. forsythia, component I did not appear to fragment, apart from the loss of NH_3_, glycine, and glycinamide (Fig. 7B).

**TABLE 3:** Accurate mass data from orbitrap MS^3^ of the *m/z* 303 ion from P. gingivalis

Peak *m/z* (*z* = 1)	Molecular formula
303.1295	C_11_H_19_N_4_O_6_
286.1030	C_11_H_16_N_3_O_6_
285.1189	C_11_H_17_N_4_O_5_
268.0925	C_11_H_14_N_3_O_5_
267.1084	C_11_H_15_N_4_O_4_
244.0925	C_9_H_14_N_3_O_5_
226.0819	C_9_H_12_N_3_O_4_
216.0975	C_8_H_14_N_3_O_4_
209.0553	C_9_H_9_N_2_O_4_
199.0710	C_8_H_11_N_2_O_4_
196.0714	C_8_H_10_N_3_O_3_
181.0605	C_8_H_9_N_2_O_3_
126.0547	C_6_H_8_NO_2_
105.0656	C_3_H_9_N_2_O_2_

10.1128/mBio.01497-20.2 FIG S2 Additional spectra (MS^4^) to determine the component I structure in P. gingivalis. Download FIG S2, PDF file, 0.3 MB. Copyright © 2020 Veith et al. 2020 Veith et al. This content is distributed under the terms of the Creative Commons Attribution 4.0 International license.

### Partial structural confirmation using hydrogen/deuterium exchange.

As noted above, the purified P. gingivalis A-LPS fragment has a protonated mass of 649 Da. The assigned structure theoretically has 13 exchangeable hydrogens which, upon exchange with deuterium, would increase the mass to 662 Da as shown in Fig. S3A. After hydrogen/deuterium exchange and MS analysis, the spectrum exhibited a distribution of peaks ranging from +6 to +13 Da due to incomplete exchange (Fig. S3A). Fragmentation of the +12-Da peak at *m/z* 661 (which is substantially more abundant than the +13) produced a major cluster of ions between *m/z* 309 and *m*/*z* 312 which were assigned to the linking sugar comprising components I and II (Fig. S3B). The nondeuterated form of this fragment has an *m/z* of 303. MS^3^ fragmentation of the *m/z* 311 peak produced component I fragments at *m/z* 109 and *m/z* 110, suggesting five exchangeable hydrogens relative to the nondeuterated form of *m/z* 105, in agreement with the proposed structure (Fig. S3C). The component II fragments were observed at *m/z* 202 and *m/z* 203, suggesting four exchangeable hydrogens relative to the nondeuterated form of *m/z* 199, also in agreement with the proposed structure (Fig. S3C).

10.1128/mBio.01497-20.3 FIG S3 Confirmation of components I and II using hydrogen/deuterium exchange MS. Download FIG S3, PDF file, 0.2 MB. Copyright © 2020 Veith et al. 2020 Veith et al. This content is distributed under the terms of the Creative Commons Attribution 4.0 International license.

### The linking sugar is partly a product of the Wbp pathway.

**FIG 9:**
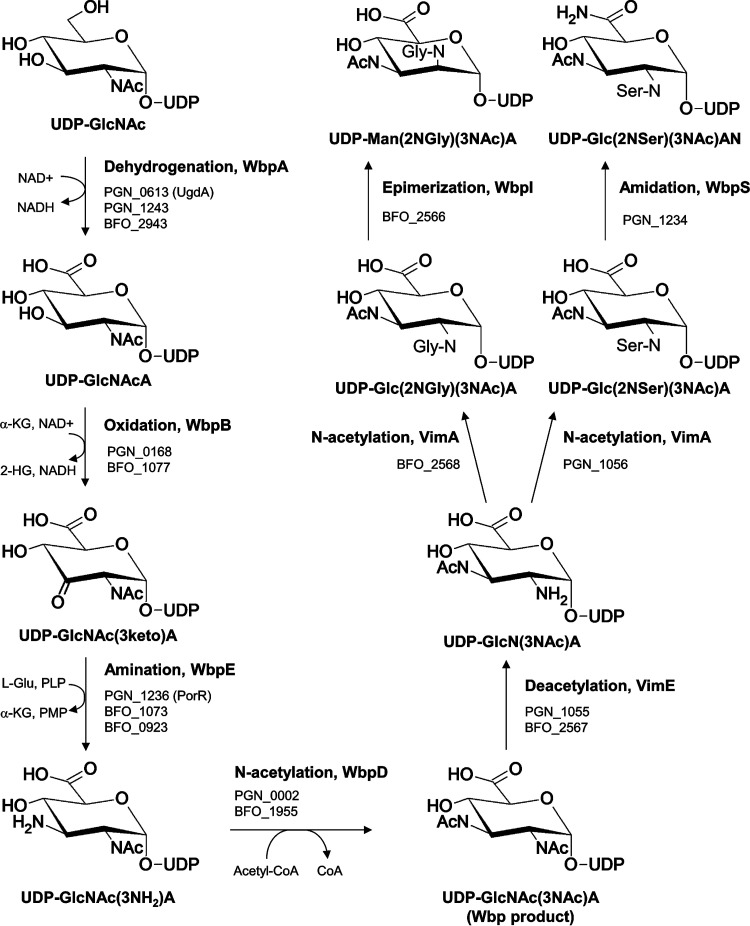
Proposed Wbp/Vim pathway for the synthesis of the linking sugars in P. gingivalis and T. forsythia. The Wbp pathway is modeled on the work previously published on P. gingivalis (18). The new additions are the steps catalyzed by VimE, VimA, and PGN_1234 (WbpS) based on the evidence presented in this study. The function of the predicted epimerase (BFO_2566) has not been experimentally tested. The sequence of the first 4 steps catalyzed by WbpA/B/E/D is well established, but it is not known whether VimA/E are dependent on the Wbp pathway. The specificities and, hence, positions in the pathway of WbpI and WbpS are also not known.

In P. gingivalis, the Wbp pathway was described to include four enzymes, WbpA, WbpB, WbpE, and WbpD, and shown to be essential for A-LPS synthesis (18). The Wbp product is expected to be a di-*N*-acetylated glucuronic acid [Fig. 9, UDP-GlcNAc(3NAc)A] similar to the linking sugars identified in this study. The only differences in P. gingivalis are seryl instead of acetyl and amide instead of acid. In T. forsythia there was only the one difference, glycyl instead of acetyl (Fig. 9, compare the Wbp product with final products). To find the genes that may be responsible for the differences in this pathway, the arrangements of the *wbp* genes in P. gingivalis and T. forsythia were compared with those of other species (Fig. S4). It was observed that a putative asparagine synthase gene (*asnB*, PGN_1234) was immediately downstream of *wbpE* (*porR*) and *wzx* (*porS*) in P. gingivalis and that a similar arrangement of genes was observed in several other species, suggesting that PGN_1234 may have a function in the Wbp pathway. Since AsnB is an amidotransferase, converting carboxylic acids to amides, we predicted that PGN_1234 may convert the glucuronic acid into glucuronamide. T. forsythia did not appear to have an *asnB* homolog, consistent with the presence of the uronic acid form.

10.1128/mBio.01497-20.4 FIG S4 Gene organization of the *wbp* locus in various species. Download FIG S4, PDF file, 0.2 MB. Copyright © 2020 Veith et al. 2020 Veith et al. This content is distributed under the terms of the Creative Commons Attribution 4.0 International license.

**FIG 10:**
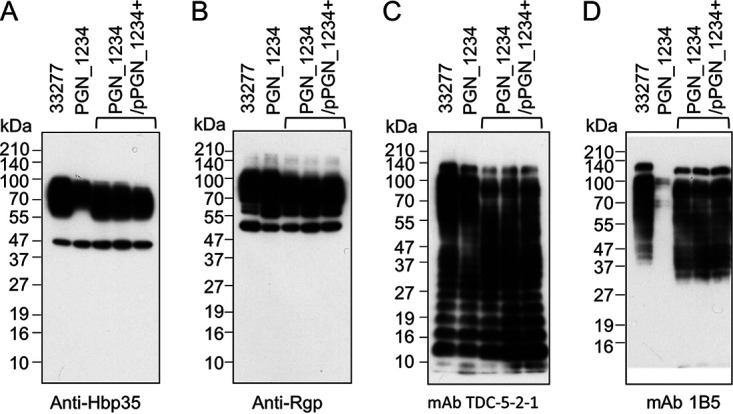
Immunoblot analysis of the *PGN_1234* mutant. Whole-cell lysates of wild-type P. gingivalis ATCC 33277, the *PGN_1234* mutant, and the complemented *PGN_1234^+^* strains were separated by SDS-PAGE and immunoblotted using various primary antibodies, as shown. MAb 1B5 detects A-LPS while MAb TDC-5-2-1 detects both A-LPS and O-LPS.

The *PGN_1234* mutant, KDP1101, was therefore constructed and characterized for its role in A-LPS modification of T9SS cargo proteins. The mutant was found to have a phenotype similar to that of the wild type (WT) in exhibiting black pigmentation and normal levels of gingipain and hemagglutination activities, consistent with normal secretion and attachment of these virulence factors to the cell surface (Fig. S5). Western blots of whole-cell lysates showed the presence of highly modified Arg gingipains and HBP35 consistent with wild type A-LPS modification (Fig. 10A and B). However, although levels of total LPS appeared normal (Fig. 10C), A-LPS was barely detected with MAb 1B5 (Fig. 10D), suggesting that PGN_1234 was required for the creation of the MAb 1B5 epitope but not essential for linkage of LPS to cargo proteins.

10.1128/mBio.01497-20.5 FIG S5 Characterization of the PGN_1234 mutant. Download FIG S5, PDF file, 0.9 MB. Copyright © 2020 Veith et al. 2020 Veith et al. This content is distributed under the terms of the Creative Commons Attribution 4.0 International license.

**FIG 11:**
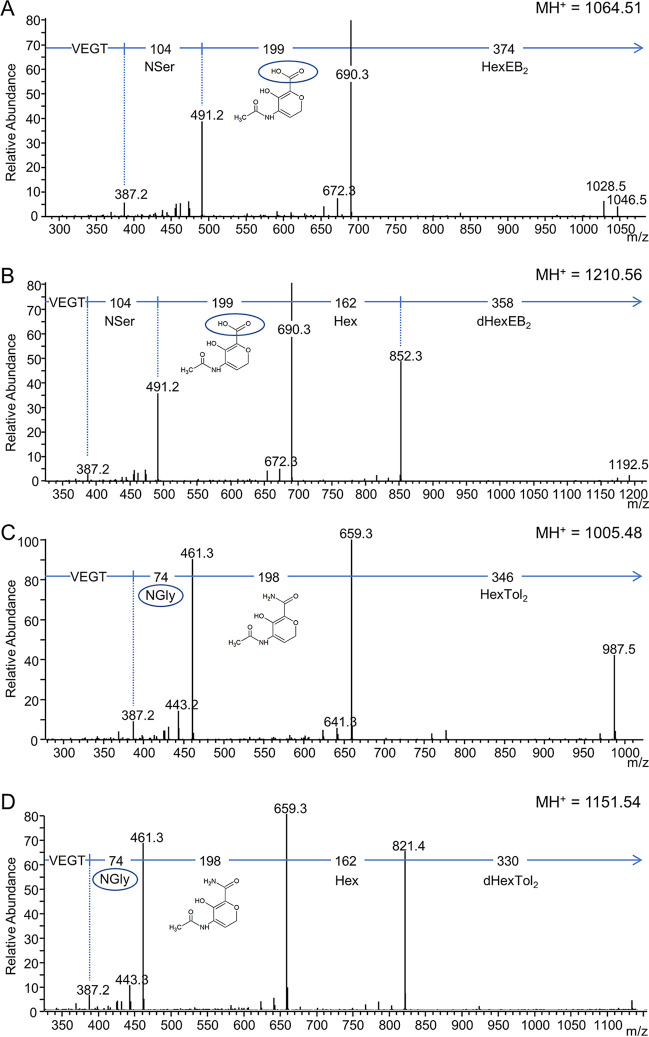
MS/MS analyses of modified RgpB peptides derived from *PGN_1234* and *vimA*_Pg_ *vimA*_Tf_*^+^* mutants. Q-Sepharose fractions that included RgpB were deglycosylated in the presence of ethylbenzene or toluene for ∼25 min. The deglycosylated samples were fractionated by SDS-PAGE, and the bands containing RgpB were digested with trypsin to produce modified C-terminal peptides with the sequence VEGT and analyzed by LC-MS/MS (CID) on an Orbitrap instrument. (A and B) MS/MS spectra derived from the *PGN_1234* mutant showing the uronic acid form rather than the amide form of component II (circled structure). (C and D) MS/MS spectra derived from the *vimA*_Pg_ *vimA*_Tf_*^+^* interspecies complementation mutant showing incorporation of glycine rather than serine. Compare to the wild-type spectra shown in Fig. 2.

To determine if PGN_1234 was responsible for glucuronamide formation, modified cargo proteins were isolated from the *PGN_1234* mutant. The fractions containing modified RgpB were subjected to deglycosylation, trypsin digestion, and analysis by LC-MS/MS. The MS/MS data revealed that component II was now 199 Da (rather than 198 Da), consistent with the inability of the mutant to convert the glucuronic acid into glucuronamide (Fig. 11A and B).

### VimA transfers the serine or glycine to complete biosynthesis of the linking sugar.

Next, we considered how the expected product of the Wbp pathway (di-*N*-acetylglucuronic acid/amide) might be converted to the observed linking sugar through the incorporation of serine or glycine. The only known proteins required for A-LPS biosynthesis that are potentially involved in sugar modifications other than those involved in the Wbp pathway are VimA and VimE. A search of the Conserved Domain database revealed that VimE is a putative member of the carbohydrate esterase 4 family which includes deacetylases (E value of 1E−83), and VimA is a putative *N*-acetyltransferase (E value of 2.9E−05). Therefore, we hypothesized that VimE may remove an acetyl group from the Wbp pathway product and that VimA may then transfer the serine (P. gingivalis) or glycine (T. forsythia) to the newly exposed amine (Fig. 9). To test this, we expressed the wild-type *vimA* gene from T. forsythia (*vimA*_Tf_*^+^*) in the P. gingivalis *vimA* mutant (*vimA*_Pg_^−^ *vimA*_Tf_*^+^*) with the expectation that T. forsythia VimA would incorporate glycine into the linker of the modified P. gingivalis cargo proteins. This strain was successfully complemented by the wild-type *vimA*_Tf_ gene, as indicated by the restoration of black pigmentation, MAb 1B5 reactivity, and the modification of cargo proteins with A-LPS (Fig. S6). The modified cargo proteins were purified from the OMVs of this complemented strain, and the RgpB fraction was subjected to deglycosylation, trypsin digestion, and analysis by LC-MS/MS, as previously described. The MS/MS data revealed that component I was now 74 Da (rather than 104 Da), consistent with the incorporation of glycine instead of serine (Fig. 11C and D). The positive MAb 1B5 reactivity to the cargo proteins despite the exchange of glycine for serine suggests that the serine side chain may not be part of the MAb 1B5 epitope. These data indicate that VimA is responsible for the specificity toward serine (P. gingivalis) or glycine (T. forsythia), and combined with the predicted *N*-acetyltransferase function, we suggest that VimA directly catalyzes this amino acid transfer.

10.1128/mBio.01497-20.6 FIG S6 The cross-species complementation with the *vimA* gene from T. forsythia can rescue the P. gingivalis *vimA*-deficient mutant. Download FIG S6, PDF file, 0.1 MB. Copyright © 2020 Veith et al. 2020 Veith et al. This content is distributed under the terms of the Creative Commons Attribution 4.0 International license.

## DISCUSSION

### Structure summary.

Through an extensive MS-based exploration, the structure of the linking sugar was concluded to be 2-*N*-seryl, 3-*N*-acetylglucuronamide in P. gingivalis and 2-*N*-glycyl, 3-*N*-acetylmannuronic acid in T. forsythia. The amino acid portions of these molecules were well supported by the MS data and by their cleavage with proteinase K. The *N*-acetyl and glucuronamide/glucuronic acid portions of the structures were also well supported, but the positions of substituents around the ring were not definitively determined. Further support from the literature for these groups is presented below. The linking sugar was bonded to a hexose residue in both species. In P. gingivalis, this hexose was further linked to both a deoxyhexose residue and C_4_H_4_O_3_ (Fig. 1).

### Novel method.

The use of TFMS is standard for the removal of glycan chains from glycoproteins leaving the protein intact (38). Generally, the cleaved glycans are not analyzed; however, a small number of studies have detected N-acetylated sugars after TFMS treatment (38). To our knowledge, this is the first study to show that simple hexoses can also be detected after TFMS treatment. As seen by other investigators, the N-acetylated sugar (component II) could be analyzed since it was not extensively cleaved and modified during the deglycosylation procedure (39). In contrast, the hexose and deoxyhexose identified in this study both reacted with the arene (toluene or EB) that was used together with TFMS. An extensive search of the literature to investigate whether this kind of chemistry had been described previously revealed that reducing sugars will react with two arenes in the presence of an AlCl_3_ catalyst under anhydrous conditions to produce 1,1-diaryl-1-deoxyalditols (40). Anhydrous AlCl_3_ is a strong Lewis acid, and TFMS is likely to be functioning in the same way. The mechanism involving AlCl_3_ proceeds through an intermediate modified by a single arene. A search of our LC-MS/MS data for this intermediate was successful (Fig. 3F), supporting the contention that the same chemistry applies to the TFMS-catalyzed reaction. The MS intensities of the peaks corresponding to the final bis-arylated product were at least 100-fold greater than the peak matching the single arylated intermediate, indicating that the reaction almost went to completion. TFMS deglycosylation can therefore be used to analyze glycan structure more broadly than previously thought.

### Relationship with published LPS structures.

The challenge these data represent is to determine how they relate to the reported A-LPS structure and therefore complete the picture of how the T9SS substrates are attached to the cell surface in P. gingivalis and T. forsythia. In P. gingivalis, A-LPS is composed of lipid A, a core oligosaccharide common to O-LPS, and a specific polysaccharide that is uniquely recognized by MAb 1B5. The outer core is composed of mannose with only some phosphoethanolamine while the inner core includes glycerol, allosamine, and KDO (3-deoxy-d-*manno*-octulosonic acid) (11). Lipid A is composed of glucosamine with N-linked and O-linked fatty acids (29). The polysaccharide of A-LPS was originally reported to be a branched phosphomannan (13). Based on these reports the structure of the A-LPS fragment reported here cannot be assigned to any known part of A-LPS. In contrast, the polysaccharide of O-LPS of P. gingivalis has been shown to have a tetrasaccharide repeating unit comprising GalNAc, Rha, Glc, and Gal (10), which represents the closest fit to our data since the Rha may be the same as the deoxyhexose identified in the unique A-LPS fragment. Consistent with this, Shoji et al. have suggested that A-LPS may contain a tetrasaccharide repeating unit that is similar to the O-polysaccharide (14). Key to this finding is the identification of the A-LPS-specific glycosyl transferases WbaP and GtfC that appear to be responsible for the addition of the first and second sugars of the A-LPS tetrasaccharide, while the glycosyltransferases GtfE and GtfB catalyze the addition of the third and fourth sugars in both O-LPS and A-LPS (14). Two further glycosyltransferases specific to A-LPS, GtfF and VimF (14, 41), potentially transfer additional sugars to form an A-LPS-specific branch. We speculate that the A-LPS fragment identified in this study corresponds to this branch; however, further work is required to determine whether the identified deoxyhexose corresponds to the Rha within the tetrasaccharide or whether the branching point is elsewhere.

In T. forsythia, to date only rough LPS (which lacks the polysaccharide component) has been isolated. The structure of the core oligosaccharide is comprised of KDO, mannose, and glucosamine (42). Since the linking sugar was not identified, the form of LPS which is bonded to cargo proteins in this species has not yet been found. Interestingly, T. forsythia has orthologs for at least three of the A-LPS-specific glycosyltransferases, including two (WbaP and GtfC) implicated in the synthesis of the P. gingivalis tetrasaccharide repeating unit (Table 4). Further work is required to elucidate the exact roles of these transferases in both species and to identify the exact attachment point of the linker to the LPS anchor.

**TABLE 4:** P. gingivalis glycosyl transferases required for A-LPS synthesis and their T. forsythia protein BLAST hits

Name^*a*^	P. gingivalis ATCC 33277	T. forsythia 92A2 top BLAST hit	Sequence identity (%)	Query cover (%)
WbaP*	PGN_1896	BFO_1803	45	98
GtfC*	PGN_0361	BFO_2130	51	99
GtfE	PGN_1240	BFO_0475	23	54
GtfB	PGN_1251	BFO_1049	28	39
VimF*	PGN_1054	BFO_2565	40	99
GtfF*	PGN_1668	BFO_1987	27	54

a Proteins marked with an asterisk are specific to A-LPS synthesis; GtfB and GtfE are required for both A-LPS and O-LPS synthesis.

### The linking sugar may be the product of the novel Wbp/Vim pathway.

Our data indicate that the linking sugar is likely to be a product of Wbp and Vim enzymes for the following reasons. Since the linking sugar connects cargo proteins to A-LPS, its biosynthesis is expected to be essential for T9SS cargo protein modification which has an absolute requirement for the gene products WbpA, WbpB, WbpD, and WbpE (18) as well as for VimA and VimE (19). Second, the elucidated structure of the linking sugar fits exactly the predicted activities of these Wbp and Vim enzymes, with WbpS being an additional nonessential enzyme utilized by P. gingivalis (Fig. 9). Third, the demonstration that PGN_1234 (WbpS) is involved in the biosynthesis of the linking sugar in P. gingivalis by converting the uronic acid into the uronamide form supports both the proposed structures and the contention that the Wbp pathway is involved in the synthesis. Finally, the proposed biosynthesis of the elucidated structures is consistent with the genetic data by accounting for all the genes known to be specific to A-LPS biosynthesis besides the glycosyltransferases (WbaP, GtfC, GtfF, and VimF). We have designated this novel linking sugar biosynthetic pathway the Wbp/Vim pathway (Fig. 9).

It follows that the linking sugar may be the only A-LPS-specific sugar required both for recognition by MAb 1B5 and for cargo modification. Indeed, the significant loss of reactivity toward MAb 1B5 observed in the *PGN_1234* mutant suggests that the uronamide of the linking sugar forms part of the MAb 1B5 epitope and is consistent with the nonreactivity of T. forsythia LPS since this species does not produce the uronamide (43). The phosphomannan was also reported to have an epitope for MAb 1B5 (13), which suggests that the phosphomannan is in close association with the linking sugar. However, both findings need to be confirmed with direct evidence. The phosphomannan may not be essential for cargo modification and the development of black pigmentation on blood agar since despite extensive screening for the genes required for black pigmentation (27), none have been found that could be specifically assigned to its biosynthesis. The phosphomannan consists of one phosphate and eight different mannoses and presumably requires several different mannosyltranferases for its synthesis, which have not been identified to date.

### Position of N-Ser and N-Gly groups.

The results presented here do not definitively show the position of any of the substituents in the linking sugar. The structure shown is the most consistent with and strongly indicated by the data, but consideration of its biosynthetic provenance strongly influenced the decision of where to place the substituents. The Wbp pathway uses a common precursor in GlcNAc, with the *N*-acetyl group in the usual C-2 position. In the second and third steps, it places an amine in the C-3 position, and then in the fourth step WbpD transfers an acetyl group to the amine (Fig. 9). The function of WbpD was successfully complemented by WbpD from Pseudomonas aeruginosa, confirming that the di-*N*-acetylglucuronic acid is formed in P. gingivalis (18, 22). It then appears that VimE removes one of the acetyl groups and that VimA transfers the amino acid. But if VimE removes the acetyl group from C-3, the product will be the same as the WbpE (PorR) product, which would seem to be an unnecessary step. Moreover, if this were the case, WbpD would not be essential for A-LPS biosynthesis. It is therefore proposed that VimE must instead target removal of the original acetyl group at C-2, consistent with our interpretation of the MS data.

### Association of *vim* genes with *wbp* genes and sortase-encoding genes in bacterial genomes.

A conserved domain search of VimA matches not only to *N*-acetyl transferases but also to the pep-cterm_femAB family (TIGR03019). This family is defined in part by its codistribution within bacterial genomes with other genes previously found to be associated with each other. These associated genes include secreted proteins having the PEP-CTERM signal, an exosortase, and genes involved in the production of exopolysaccharide (EPS) (44). The PEP-CTERM signal includes the PEP motif, a putative transmembrane helix, and a positively charged C terminus. After translocation across the inner membrane (IM), the exosortase which is embedded in the IM is proposed to cleave near the PEP motif, leaving the transmembrane helix in the IM and conjugating the new C terminus to an unidentified compound. The protein is then expected to be secreted across the OM by an unknown pathway and become associated with the EPS (44). It is therefore interesting to speculate that the VimA-related proteins in these bacteria may be involved in producing the unidentified compound which may also be an amino acid-modified sugar. To investigate this further, some of the genetic loci containing these VimA-related proteins were examined (see Fig. S7 in the supplemental material). In Nitrosomonas eutropha, the *vimA*-related gene was adjacent to a polysaccharide deacetylase, glycosyltransferases, exosortase (EpsH), and a gene annotated as asparagine synthase, which matched to PGN_1234 by protein PSI-BLAST. A similar gene arrangement was observed in Nitrosococcus oceani and Desulfovibrio vulgaris (Fig. S7). Conserved domain searches of the two asparagine synthases produced top matches to the eps_aminotran_1 family (TIGR03108), which represents another protein associated with the PEP-CTERM system. To further understand the gene arrangement of *vimA*, *vimE*, and *PGN_1234* homologs, PSI-BLAST searches were conducted for the three proteins, and the gene arrangements in some of the most prominently matching species were briefly examined. In Pseudomonas mosselii, Aeromonas veronii, and Vibrio cholerae, *vimA* homologs were found adjacent to *vimE* homologs, and genes belonging to the complete Wbp pathway were also found in the same cluster. These species do not have exosortases to our knowledge, but V. cholerae and A. veronii both have a rhombosortase (Fig. S7). Rhombosortases are associated with secreted proteins with the GlyGly-CTERM motif (45). Rhombosortase from V. cholerae has been shown experimentally for one protein to cleave at the GG motif and conjugate the C terminus to a moiety deduced to contain glycerophosphoethanolamine, which is required for cell surface anchorage after secretion through a type II secretion system (T2SS) (46). Other CTERM systems have also been identified in other bacteria (47). Further work is required to determine if the Wbp/Vim pathway is used for protein modification and cell surface attachment in any of these CTERM systems.

10.1128/mBio.01497-20.7 FIG S7 Arrangement of selected genetic loci containing VimA-related proteins. Download FIG S7, PDF file, 0.5 MB. Copyright © 2020 Veith et al. 2020 Veith et al. This content is distributed under the terms of the Creative Commons Attribution 4.0 International license.

The PSI-BLAST searches of PGN_1234 revealed that it was more closely related to WbpS in P. aeruginosa and its homolog WbqG in Escherichia coli O121, both members of the eps_aminotran_1 family, than to other AsnB-related proteins in the same species (Table S3). WbpS has long been proposed to convert *N*-acetyl galacturonic acid into the uronamide form (48), and its homolog, WbqG, was demonstrated to be required for this activity (49). We therefore propose the name WbpS for PGN_1234.

## MATERIALS AND METHODS

### Bacterial strains, plasmids, and bacterial growth.

Bacterial strains and plasmids used in this study are listed in Table S1 in the supplemental material. P. gingivalis strains were grown on solid medium containing Trypticase soy agar (40 g/liter), brain heart infusion broth (5 g/liter), 5% (vol/vol) lysed defibrinated horse blood, cysteine hydrochloride (0.5 g/liter), and menadione (5 mg/ml) (TSBHI agar) or in tryptic soy (TS)-enriched brain heart infusion broth (TSBHI) (25 g/liter tryptic soy, 30 g/liter BHI broth) supplemented with 0.5 mg/ml cysteine, 5 μg/ml hemin, and 5 μg/ml menadione, both under anaerobic conditions (80% N_2_, 10% H_2_, and 10% CO_2_) at 37°C. T. forsythia was grown as previously described (37). Cells were harvested by centrifugation at 8000 × *g*. Luria-Bertani (LB) broth, and LB agar plates were used for growth of Escherichia coli strains. Antibiotics were used at the following concentrations: ampicillin (Ap; 100 μg/ml for E. coli and 10 μg/ml for P. gingivalis), erythromycin (Em; 10 μg/ml for P. gingivalis), gentamicin (Gm; 50 μg/ml for wild-type P. gingivalis), and tetracycline (Tc; 0.7 μg/ml for P. gingivalis).

10.1128/mBio.01497-20.8 TABLE S1 Bacterial strains and plasmids used in this study. Download Table S1, DOCX file, 0.04 MB. Copyright © 2020 Veith et al. 2020 Veith et al. This content is distributed under the terms of the Creative Commons Attribution 4.0 International license.

### Construction of the *PGN_1234* mutant.

All DNA primer sequences used in this study are listed in Table S2. The P. gingivalis *PGN_1234* mutant was constructed by removal of the coding region corresponding to E^71^-L^542^ by making a suicidal plasmid with *PGN_1234* upstream and downstream regions either side of an *ermF* antibiotic resistance cassette (pKD1401). This was done by exchange of upstream and downstream regions of the plasmid that was used for deletion of the *hbp35* gene (pKD740, *hbp35*::*ermF* in pGEM-T Easy plasmid) (50). All PCRs used PrimeSTAR Max DNA polymerase (TaKaRa, Japan). When amplicons were cloned into pUC118, a HincII/BAP-treated vector (where BAP is bacterial alkaline phosphatase) and Mighty Cloning Reagent Set from TaKaRa were used. The PGN_1234 upstream and downstream regions were separately amplified by PCR using the primer pair PGN1234upFw and PGN1234upRev and the pair PGN1234dwFw and PGN1234dwRev, respectively, and P. gingivalis ATCC 33277 genomic DNA as the template. The upstream amplicon was cloned into pUC118 to produce pUC118-*PGN_1234*_up_. The downstream amplicon was cloned into pUC118 to produce pUC118-*PGN_1234*_dw_. The *PGN_1234* upstream region was cleaved and purified from pUC118-*PGN_1234*_up_ using SphI-BamHI and ligated into the SphI-BamHI-cleaved *hbp35* deletion plasmid pKD740 for upstream region exchange, producing p*PGN_1234*_up_-*ermF*-*hbp35*_dw_. The *PGN_1234* downstream region was cleaved and purified from pUC118-*PGN_1234*_dw_ using PstI-SacI and ligated into PstI-SacI-cleaved p*PGN_1234up*-*ermF*-*hbp35*_dw_ for downstream region exchange producing the *PGN_1234* deletion plasmid, pKD1401 (p*PGN_1234*_up_-*ermF*-*PGN_1234*_dw_). Finally, pKD1401 was linearized with SphI and introduced into P. gingivalis ATCC 33277 by electroporation and selection on blood agar plates containing 10 μg/ml erythromycin to obtain the Em-resistant transformant KDP1101.

10.1128/mBio.01497-20.9 TABLE S2 Primers used in this study. Download Table S2, PDF file, 0.01 MB. Copyright © 2020 Veith et al. 2020 Veith et al. This content is distributed under the terms of the Creative Commons Attribution 4.0 International license.

10.1128/mBio.01497-20.10 TABLE S3 PGN_1234 PSI-BLAST matches to WbpS and WbqG compared to AsnB Download Table S3, PDF file, 0.04 MB. Copyright © 2020 Veith et al. 2020 Veith et al. This content is distributed under the terms of the Creative Commons Attribution 4.0 International license.

### Construction of the complemented *PGN_1234* mutant.

To create the *PGN_1234* complementation E. coli-P. gingivalis shuttle plasmid, first, the coding region of the *porK*^+^ complementation plasmid, pBSSK-p-*porK^+^-*T (pKD955) (28), was replaced with the *PGN_1234* coding region. This construct allows transcription of the coding region to be driven by the Porphyromonas gulae catalase gene promoter (p) and terminated by the P. gingivalis ATCC 33277 *rgpB* terminator (T). The *PGN_1234* coding region was amplified using the primer pair PGN1234compFw and PGN1234compRv and P. gingivalis ATCC 33277 genomic DNA as the template. The amplicon was cloned into pUC118 yielding pUC118-*PGN_1234^+^*. The *porK* coding region of pBSSK-p-*porK^+^*-T (pKD955) was cleaved by SalI-XbaI and replaced with the SalI-XbaI-cleaved *PGN_1234* coding region from pUC118-*PGN_1234^+^* to yield pBSSK-p-*PGN_1234^+^*-T. This plasmid was then cleaved with KpnI-NotI, and the DNA fragment containing p-*PGN_1234^+^*-T was ligated into a KpnI-NotI-cleaved E. coli-P. gingivalis shuttle plasmid, pTCB (51), to yield the *PGN_1234* complementation plasmid, pTCB-*PGN_1234^+^*. This complementation plasmid was introduced into Escherichia coli S17-1 (52) by electroporation. The resulting E. coli transformant was conjugated with the P. gingivalis ATCC 33277 *PGN_1234* deletion mutant, KDP1101. Transconjugants were selected on blood agar plates containing Gm (50 μg/ml) and Tc (0.7 μg/ml), yielding the *PGN*_*1234*::*ermF* pTCB-*PGN_1234^+^* strain (KDP1102).

### Interspecies *vimA* complementation.

A complementation plasmid that replaces the P. gingivalis C-terminal region of *mfa1* and N-terminal region of *mfa2* genes with the *vimA* gene (BFO_2568) of Tannerella forsythia was constructed. First, the N-terminal coding region of the *mfa1* gene (*mfa1*_N_) was amplified by PCR using the primer pair mfa1-F and mfa1-R and P. gingivalis ATCC 33277 genomic DNA as the template. The amplicon was cloned into pUC118 producing pUC118-*mfa1*_N_ and was confirmed by DNA sequencing. The C-terminal coding region of the *mfa2* gene (*mfa2*_C_) was amplified by PCR using the primer pair mfa2-F and mfa2-R and P. gingivalis ATCC 33277 genomic DNA as the template. The amplicon was cloned into pUC118 and the orientation with the primer BamHI site closest to the vector SphI site was confirmed by DNA sequencing producing pUC118-*mfa2*_C_. The pUC118-*mfa2*_C_ was digested by SacI and self-ligated to eliminate the pUC118 BamHI site, producing pUC118-*mfa2*_C_2_. The SphI-BamHI-cleaved *mfa1*_N_ DNA fragment from pUC118-*mfa1*_N_ was then ligated into the SphI-BamHI sites of pUC118-*mfa2*_C_2_ producing pUC118-*mfa1*_N_-*mfa*2_C_. The BamHI-PstI DNA fragment encoding the *ermF* gene from pAL30 was inserted into the BamHI-PstI sites of pUC118-*mfa1*_N_-*mfa2*_C_, producing p*mfa1*_N_*-ermF-mfa2*_C_. The PCR fragment containing the P. gulae catalase promoter fused to the *T. forsythia vimA* coding sequence (BFO_2568) was generated as follows. The promoter region of the *P. gulae* catalase gene (p) from pKD954 (28) was amplified by PCR with the primers Pcat-F and Pcat-R. The T. forsythia *vimA* gene was amplified by PCR with the primers TfvimA-F and TfvimA-R and genomic DNA from *T. forsythia* ATCC 43037. The promoter and *vimA* amplicons (with a 16 nucleotide-overlap between them) were purified and used as PCR template with the primer pair Pcat-F and TfvimA-R, primer pair producing the p-*vimA*_Tf_*^+^* gene fusion. This amplicon was cloned into pUC118, producing pUC118-p-*vimA*_Tf_*^+^* and confirmed by DNA sequencing. The p-*vimA*_Tf_*^+^*-containing KpnI-NotI DNA fragment from pUC118-p-*vimA*_Tf_*^+^* was ligated into KpnI-NotI-cleaved p*mfa1*_N_*-ermF-mfa2*_C_, producing the *vimA*_Tf_*^+^* complementation plasmid, p*vimA*_Tf_*^+^* (pUC118-*mfa1*_N_*-ermF-*p-*vimA*_Tf_*^+^-mfa2*_C_). This plasmid was linearized with SphI, purified, and introduced into the P. gingivalis ATCC 33277 *vimA* mutant, KDP202 (*vimA*::*tetQ*) (53) by electroporation. The complemented recombinant (KDP1103, ATCC 33277 *vimA*::*tetQ*, *mfa1*_N_ *mfa2*_C_::*ermF*-p-*vimA*_Tf_*^+^*) was selected on TS agar containing 10 μg/ml Em and incubated under anaerobic conditions for 7 days. Similarly, p*mfa1*_N_*-ermF-mfa2*_C_ was linearized and introduced into the ATCC 33277 *vimA* mutant by electroporation and a selected transformant (KDP1104, ATCC 33277 *vimA*::*tetQ*, *mfa1*_N_ *mfa2*_C_::*ermF*) was used as a control.

### Fractionation and protein purification.

Outer membrane vesicles (OMVs) were prepared as previously described for P. gingivalis (54) and T. forsythia (37). Modified cargo proteins of P. gingivalis strains were purified from OMVs isolated from cultures grown for 3 to 6 days. The OMVs were solubilized in 1% myristyl sulfobetaine (SB3-14; Sigma-Aldrich), and the proteins were separated by anion-exchange chromatography using a Q-Sepharose column equilibrated in 20 mM Bis-Tris, 5 mM CaCl_2_, 50 mM NaCl, and 0.05% SB3-14, pH 6, and eluted with a linear gradient of 50 to 500 mM NaCl. Q-Sepharose fractions containing modified cargo proteins were combined and concentrated using a 10-kDa-molecular-weight cutoff membrane.

### Deglycosylation and digestion with trypsin or proteinase K.

T. forsythia OMVs or purified proteins were precipitated using 10% trichloroacetic acid (TCA), and the pellet was resuspended in 50% acetonitrile–0.1% aqueous trifluoroacetic acid. The samples were transferred to glass vials and freeze-dried thoroughly. Deglycosylation was performed using the protocol from the manufacturer of the PROzyme/Glyko Glycofree chemical deglycosylation kit (GKK-500). Briefly, samples were deglycosylated in 90% TFMS (catalog not. 347817; Sigma) and 10% anhydrous toluene or anhydrous ethylbenzene (EB) at −20°C for 4 h or the time indicated in Table 1 and figure legends. The reaction mixture was slowly neutralized on an ethanol-dry ice bath with three volumes of pyridine-methanol-water at a ratio of 3:1:1. Ammonium bicarbonate (50 mM, 8 vol) was then added. The deglycosylated cargo proteins were recovered by TCA precipitation and analyzed by SDS-PAGE. In-gel digestion on the protein bands was performed with trypsin as described previously (36). Proteinase K digestion (1 μg of proteinase K per band) was performed on the tryptic fragments at 45°C for 4 h.

### Purification of LPS fragments.

Proteinase K-digested samples were purified by off-line high-performance liquid chromatography (HPLC) using an UltiMate 3000 system fitted with a precolumn of PepMap C_18_(300-μm inner diameter by 5 mm) and an analytical column of PepMap C_18_ (300-μm inner diameter by 15 cm) (both from ThermoFisher). Fractions close to the expected retention (based on LC-MS/MS analysis) were collected manually, and a small portion was analyzed by LC-MS/MS on an HCT Ultra ion trap (see below). Fractions containing the desired LPS fragments (at *m/z* 649 or 620) were pooled and stored at –20°C.

### SDS-PAGE and immunoblotting.

SDS-PAGE for deglycosylation experiments was performed using 10% polyacrylamide Bis-Tris gels with morpholinepropanesulfonic acid (MOPS) buffer (Invitrogen/Thermo Fisher Scientific) under reducing conditions and stained with Coomassie blue. Immunoblotting was performed as described previously (53) using MAb 1B5, which recognizes the anionic polysaccharide of A-LPS and which was kindly provided by M. A. Curtis (15), MAb TDC-5-2-1, which detects both A-LPS and O-LPS (43, 55), anti-HBP35 (56), and anti-Rgp (43).

### Enzyme activity assays.

Hemagglutination and proteolytic activities were determined as previously described (43).

### Hydrogen/deuterium exchange.

The purified A-LPS fragment of P. gingivalis (*m/z* 649) was freeze-dried, redissolved in 50% D_2_O–50% CH_3_OD containing 0.1 M CH_3_CO_2_D, completely dried again, and redissolved in the same solvent (57).

### Bioinformatics.

The Metlin database at https://www.metlin.scripps.edu/ was used to find known structures that match the accurate mass data. Molecular formulae were generated from accurate mass data using the MF Finder tool at https://www.chemcalc.org. T. forsythia MS/MS data were searched using Mascot as previously described (37) with the addition of new optional C-terminal modifications as described in the results.

### Direct infusion MS.

Multistage mass spectrometry experiments were performed using two different instruments. The first was a hybrid LTQ and 7 T Fourier transform ion cyclotron resonance mass spectrometer (LTQ-FTICR; Thermo, Bremen, Germany). In general, ions were mass selected using an isolation width of 1.5 to 2 *m/z* and subjected to collision-induced dissociation (CID) using a normalized collision energy (NCE) of 30. Where possible, ion formulae were confirmed via accurate mass measurements in the FTICR cell. The collision gas was helium in all cases, and the instrument was calibrated with the recommended calibration solution consisting of caffeine, the tetrapeptide MRFA, and Ultramark 1621. The second instrument was an Orbitrap Fusion Lumos Tribrid mass spectrometer (Thermo Fisher Scientific, San Jose, CA, USA). CID-MS^n^ spectra were acquired using a mass resolution of 120,000 (at *m/z* 200), an isolation width of 1 *m/z*, an auto-gain control target of 1e4, an NCE of 30, and an *m/z* range from 50 to 300. In both cases, samples were introduced into the instrument using an Advion Triversa NanoMate nano-electrospray ionization source (Advion, Ithaca, NY, USA) with a spray voltage of 1.4 kV and a gas pressure of 0.3 lb/in^2^.

### LC-MS/MS.

LC-MS/MS was conducted using an Ultimate 3000 nano-LC system connected to either an LTQ Orbitrap Elite (Thermo) or Esquire HCT Ultra ion trap (Bruker), both as previously described (36).
