# Supplementary material for: Type IX Secretion System Cargo Proteins Are Glycosylated at the C Terminus with a Novel Linking Sugar of the Wbp/Vim Pathway
Source: mBio. 2020 Sep 1;11(5):e01497-20. doi: 10.1128/mBio.01497-20 (PMC7468200; doi:10.1128/mBio.01497-20)
Supplement: FIG S1 [file mBio.01497-20-sf001.pdf]

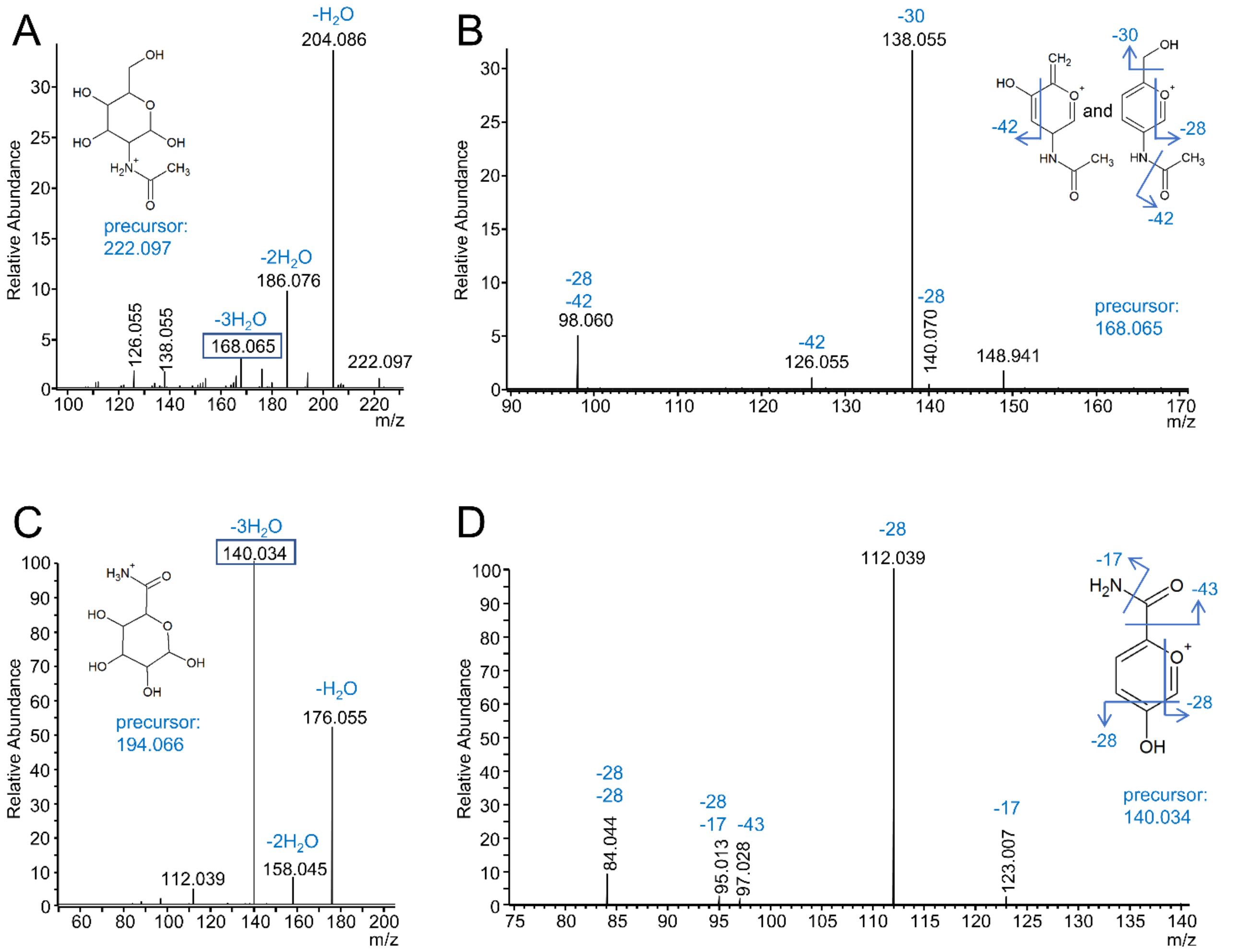


**Figure S1: MS$^n$ of N-acetyl glucosamine and glucuronamide.** A, MS$^2$ spectrum of N-acetyl glucosamine showing losses of one, two and three water molecules. The boxed product ion (*m/z* 168.065) was targeted for MS$^4$ analysis. B, MS$^4$ spectrum of *m/z* 168 (222.0900@cid30.00 204.0800@cid30.00 168.0600@cid30.00). C, MS$^2$ spectrum of glucuronamide showing losses of one, two and three water molecules. The boxed product ion (*m/z* 140.034) was targeted for MS$^3$ analysis. D, MS$^3$ spectrum of *m/z* 140 (194.0600@cid50.00 140.0340@cid25.00). The proposed structure and fragmentation of the dehydrated parent ions are shown. Both spectra were acquired by direct infusion into the Orbitrap Lumos instrument.
