# Supplementary material for: Type IX Secretion System Cargo Proteins Are Glycosylated at the C Terminus with a Novel Linking Sugar of the Wbp/Vim Pathway
Source: mBio. 2020 Sep 1;11(5):e01497-20. doi: 10.1128/mBio.01497-20 (PMC7468200; doi:10.1128/mBio.01497-20)
Supplement: FIG S2 [file mBio.01497-20-sf002.pdf]

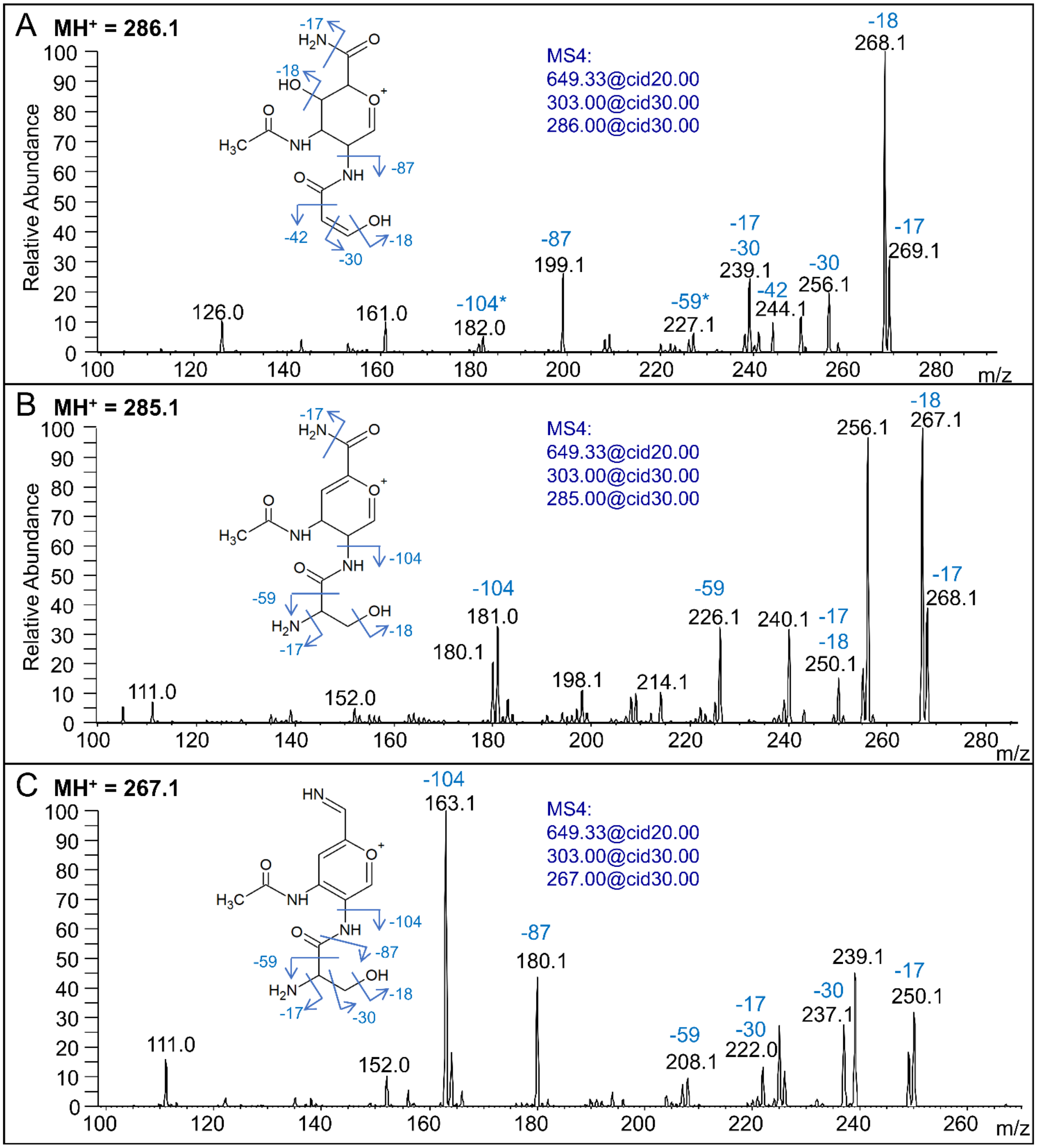

Figure S2. Additional spectra (MS^4) to determine the component I structure in *P. gingivalis.* The proposed structure and fragmentation of the precursor ions are shown. All spectra were acquired by direct infusion into the FTICR instrument.
