# Supplementary material for: Type IX Secretion System Cargo Proteins Are Glycosylated at the C Terminus with a Novel Linking Sugar of the Wbp/Vim Pathway
Source: mBio. 2020 Sep 1;11(5):e01497-20. doi: 10.1128/mBio.01497-20 (PMC7468200; doi:10.1128/mBio.01497-20)
Supplement: FIG S3 [file mBio.01497-20-sf003.pdf]

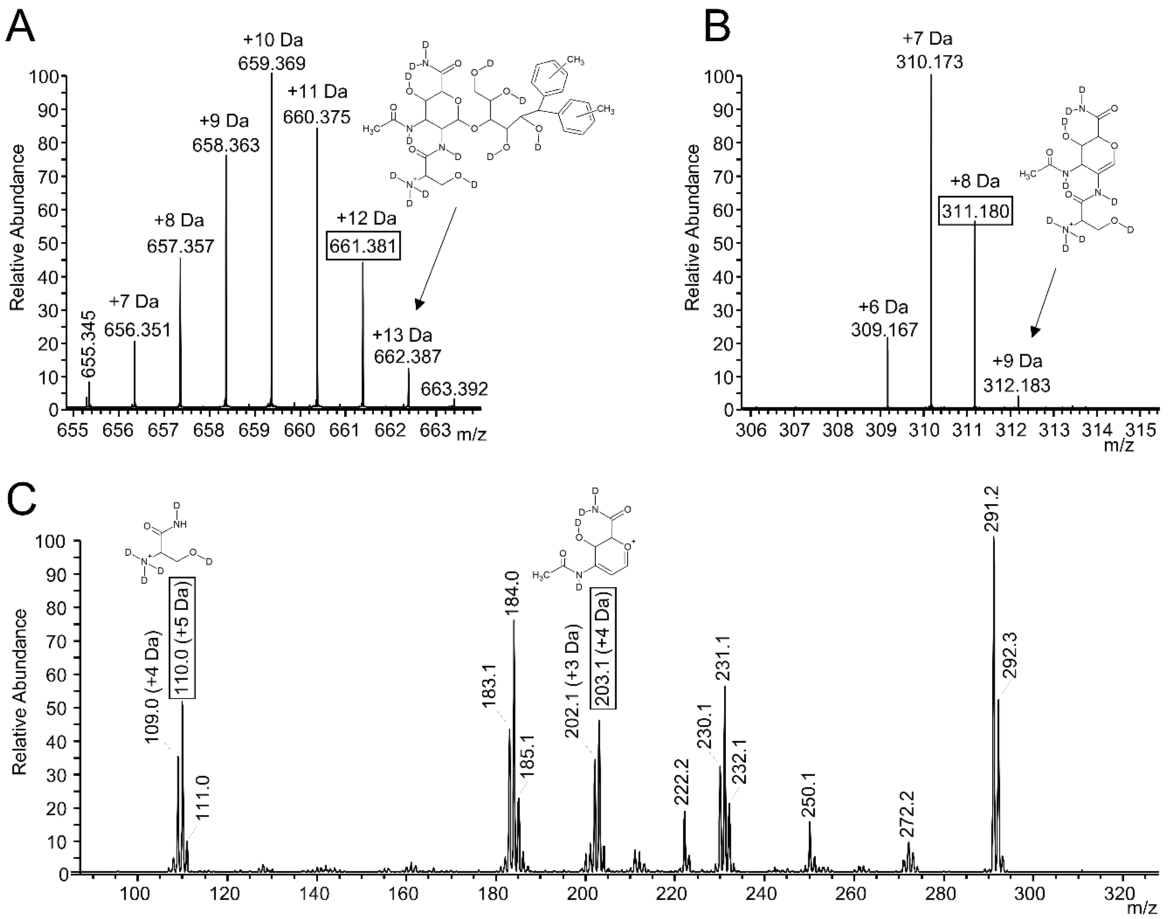

Figure S3: Confirmation of components I and II using hydrogen/deuterium exchange MS. A, MS spectrum of purified A-LPS fragment from *P. gingivalis* W50 after hydrogen/deuterium exchange. The boxed product ion was targeted for MS^2 analysis. B, MS^2 spectrum of *m/z* 661. The boxed product ion was targeted for MS^3 analysis. C, MS^3 spectrum of *m/z* 311 (661.40@cid30.00 311.30@cid30.00). The structures shown represents the fully deuterated (MD^+) forms of components I (110 Da), II (203 Da), I-II (312 Da), & I-II-III (662 Da). Note that one H is bonded to N in the fully deuterated form of component I. This is because the origin of the H is from C4 of the sugar and therefore not exchangeable.
