# Supplementary material for: Type IX Secretion System Cargo Proteins Are Glycosylated at the C Terminus with a Novel Linking Sugar of the Wbp/Vim Pathway
Source: mBio. 2020 Sep 1;11(5):e01497-20. doi: 10.1128/mBio.01497-20 (PMC7468200; doi:10.1128/mBio.01497-20)
Supplement: FIG S4 [file mBio.01497-20-sf004.pdf]

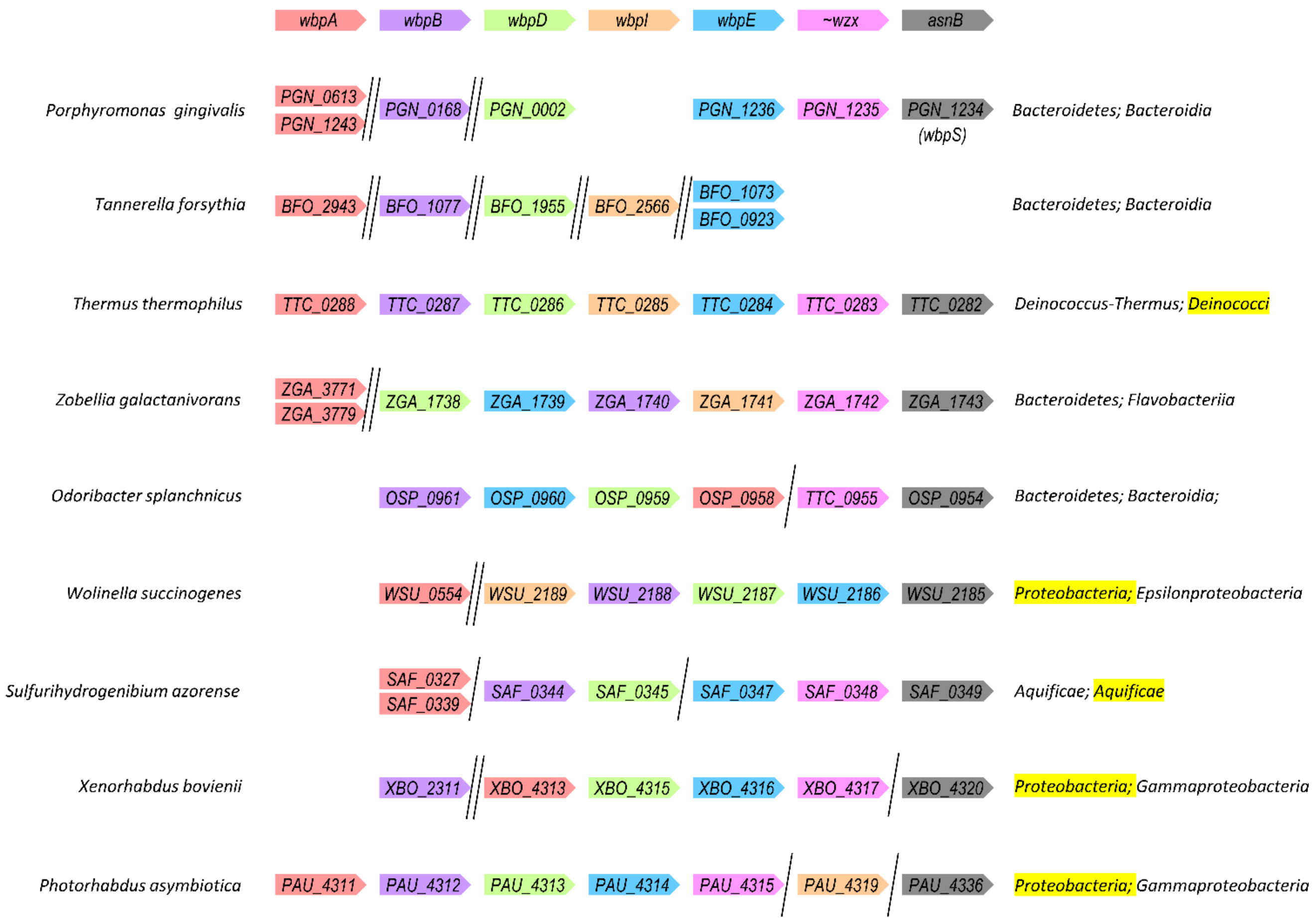

Figure S4. Gene organization of the *wbp* locus in various species. The species is indicated on the left, and the *Phylum; Class* taxonomic designations are shown on the right hand side. Genes are color-coded according to the scheme shown on the top row. Genes are directly adjacent unless separated by a single slash (nearby gene) or double slash (distant gene).
