# Supplementary material for: Type IX Secretion System Cargo Proteins Are Glycosylated at the C Terminus with a Novel Linking Sugar of the Wbp/Vim Pathway
Source: mBio. 2020 Sep 1;11(5):e01497-20. doi: 10.1128/mBio.01497-20 (PMC7468200; doi:10.1128/mBio.01497-20)
Supplement: FIG S5 [file mBio.01497-20-sf005.pdf]

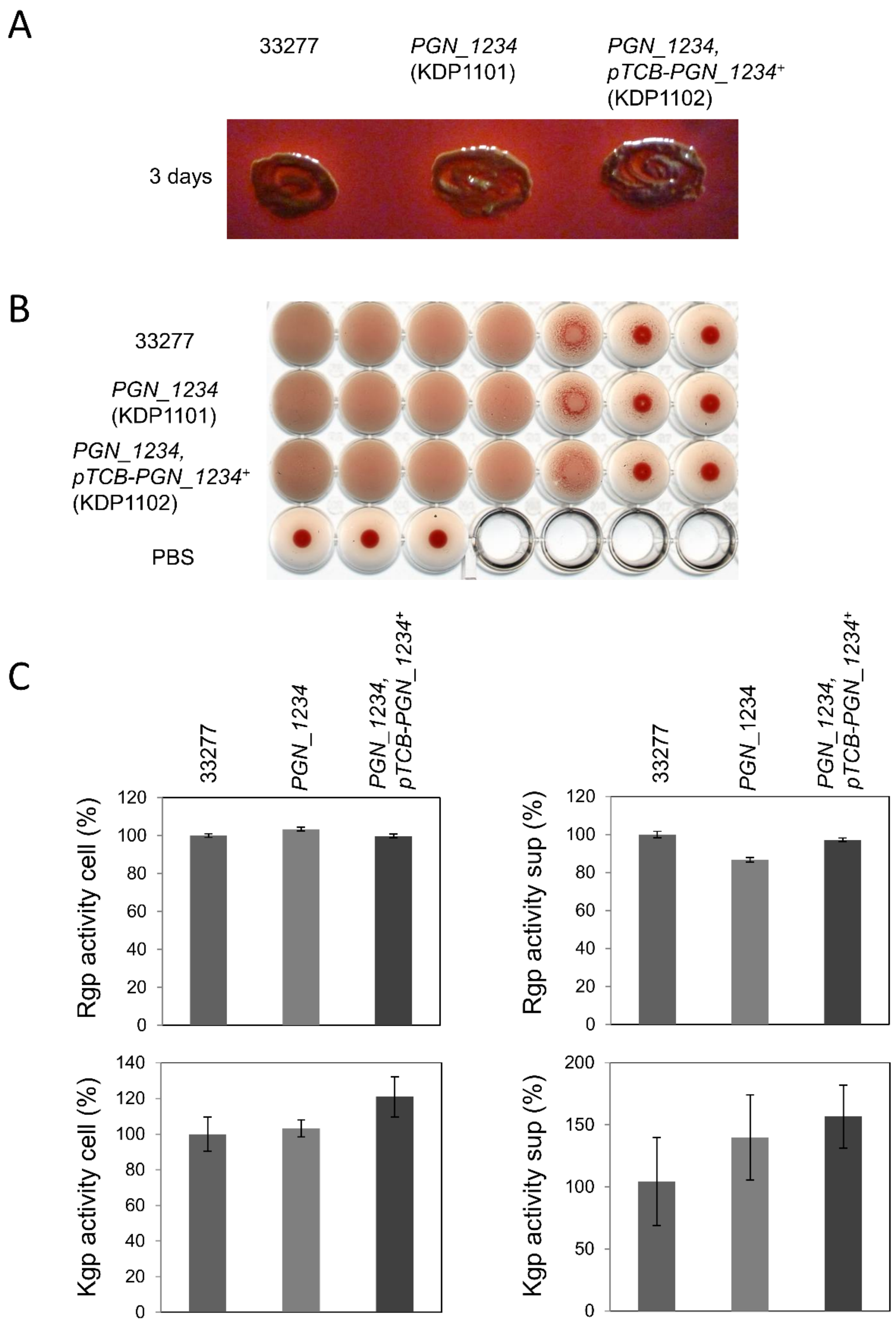

Figure S5. Characterisation of PGN_1234 mutant

A. Black pigmentation of *P. gingivalis* strains grown 3 days on blood agar. **B.** Hemagglutination assay for *P. gingivalis* strains mixed with defibrinated sheep red blood cells. **C.** Cell surface (cell) and culture supernatant (sup) Rgp and Kgp gingipain proteolytic activities relative the ATCC 33277 wild type strain.
