# Supplementary material for: Type IX Secretion System Cargo Proteins Are Glycosylated at the C Terminus with a Novel Linking Sugar of the Wbp/Vim Pathway
Source: mBio. 2020 Sep 1;11(5):e01497-20. doi: 10.1128/mBio.01497-20 (PMC7468200; doi:10.1128/mBio.01497-20)
Supplement: FIG S6 [file mBio.01497-20-sf006.pdf]

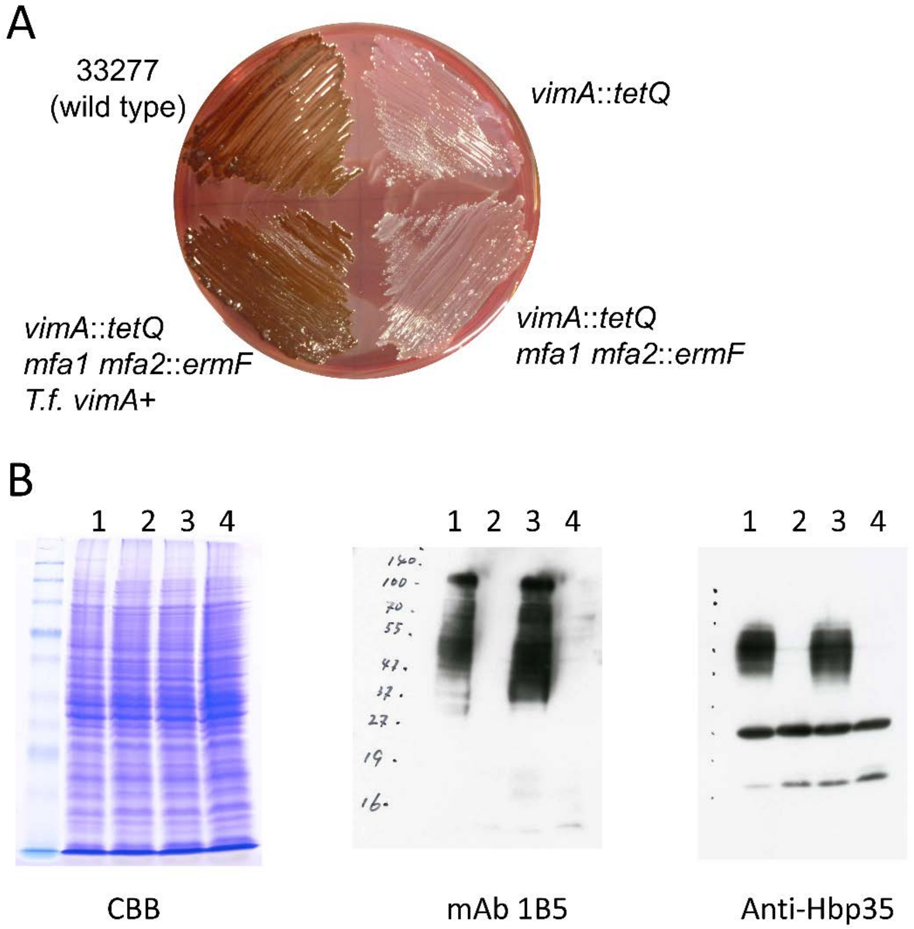

Figure S6. The cross species complementation with the *vimA* gene from *T. forsythia* can rescue the *P. gingivalis vimA* deficit mutant. A. Colony pigmentation after 3 days of growth. **B.** SDS PAGE (10%) of whole cell lysates of *P. gingivalis* strains and stained with Coomassie Blue (CBB) or subjected to Western blot (mAb 1B5 or anti-Hbp35). Lane 1, ATCC 33277; lane 2. KDP202 (*vimA*::*tetQ*); lane 3, KDP1103 (*vimA*::*tetQ mfa1Nmfa2C*::*ermF*-p-*vimA*(Tf)$^{+}$); lane 4, KDP1104 (*vimA*::*tetQ mfa1Nmfa2C*::*ermF*).
