# Supplementary material for: Type IX Secretion System Cargo Proteins Are Glycosylated at the C Terminus with a Novel Linking Sugar of the Wbp/Vim Pathway
Source: mBio. 2020 Sep 1;11(5):e01497-20. doi: 10.1128/mBio.01497-20 (PMC7468200; doi:10.1128/mBio.01497-20)
Supplement: FIG S7 [file mBio.01497-20-sf007.pdf]

Figure S7. Arrangement of selected genetic loci containing VimA-related proteins.
This figure includes the following 6 pages.

Nitrosomonas eutropha C91

Nitrosomonas eutropha C91, complete genome

GenBank: CP000450.1

GenBank FASTA

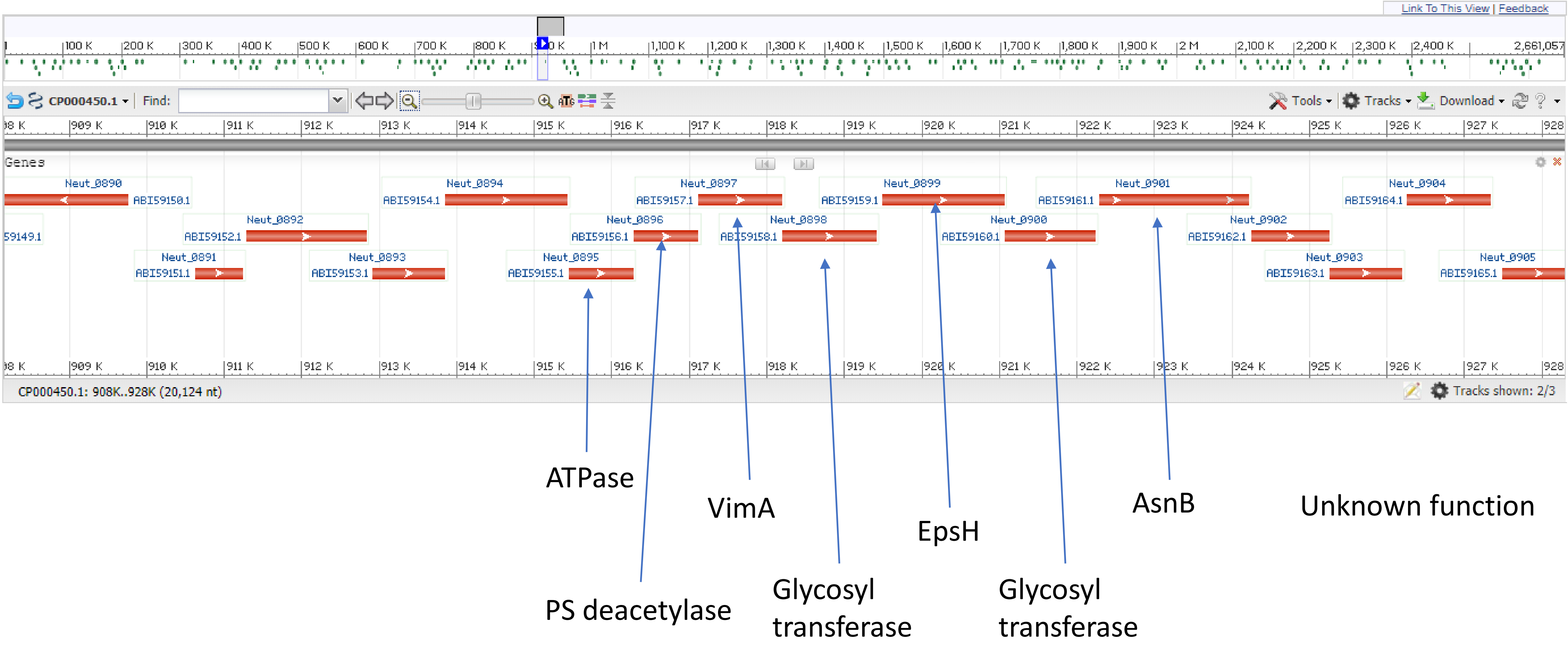

https://www.ncbi.nlm.nih.gov/nuccore/CP000450.1?report=graph&log$=seqview

Nitrosococcus oceani ATCC 19707, complete genome

GenBank: CP000127.1

GenBank FASTA

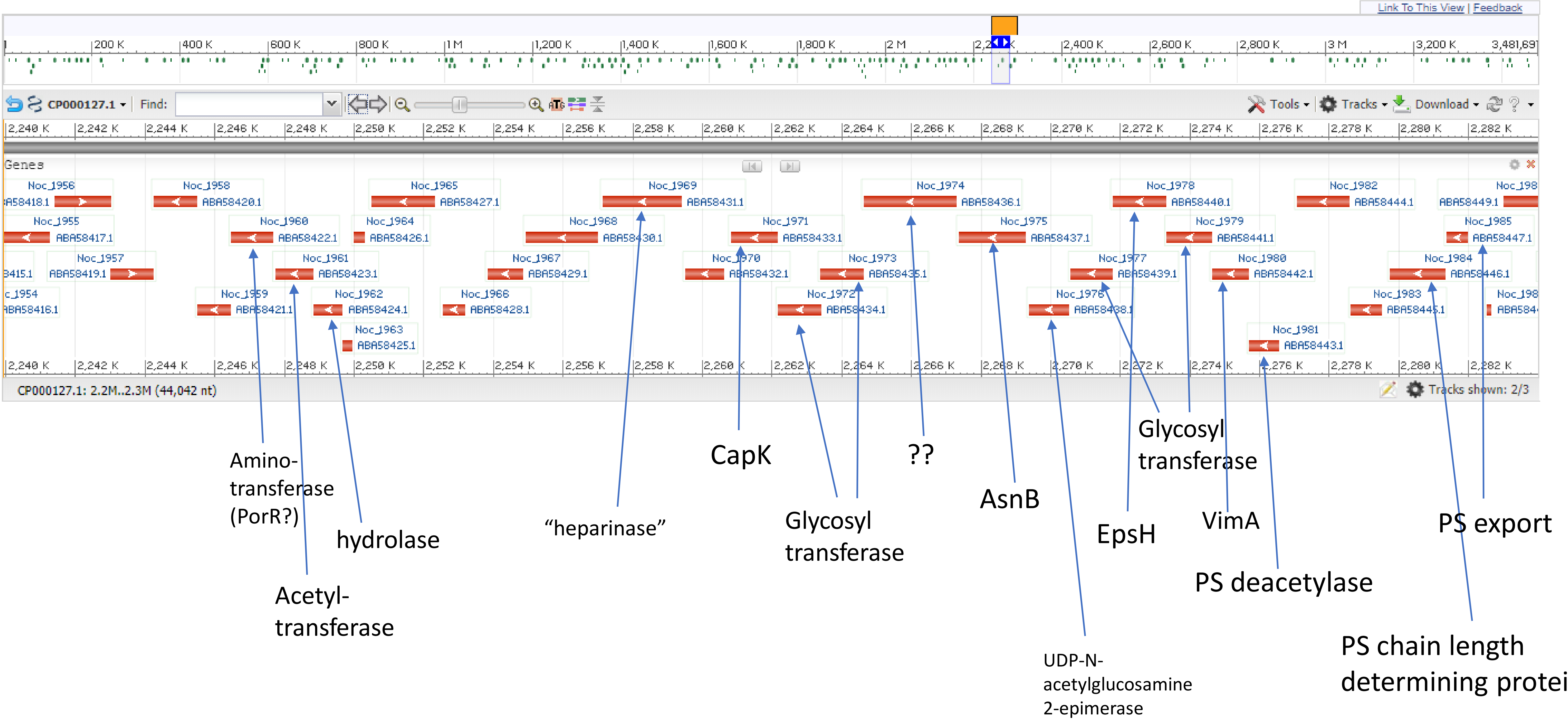

https://www.ncbi.nlm.nih.gov/nuccore/CP000127.1?report=graph&log$=seqview

Desulfovibrio vulgaris DP4 plasmid pDVUL01, complete sequence

GenBank: CP000528.1

GenBank FASTA

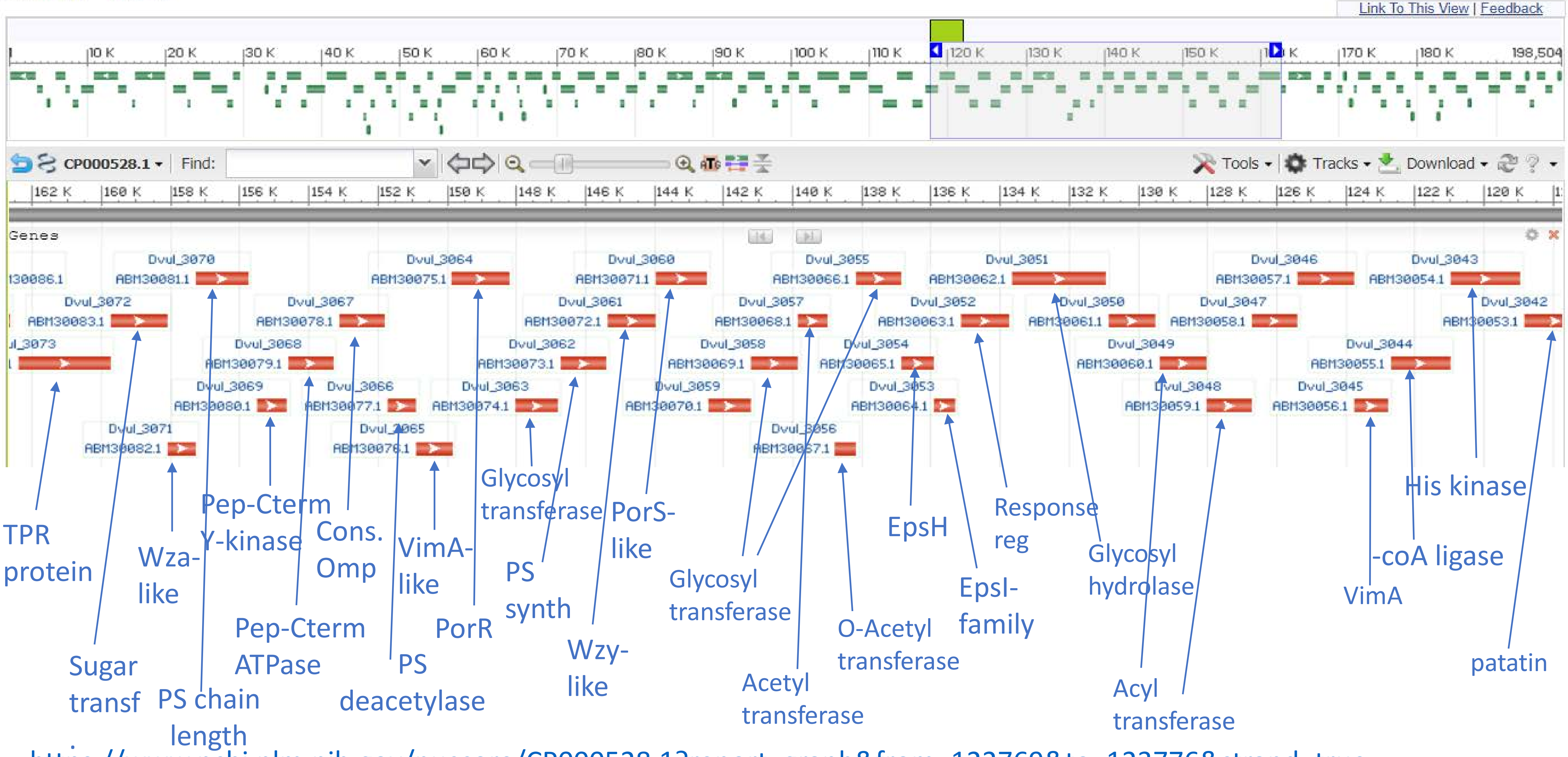

https://www.ncbi.nlm.nih.gov/nuccore/CP000528.1?report=graph&from=122769&to=123776&strand=true

Pseudomonas mosselii strain SC006 Scaffold4

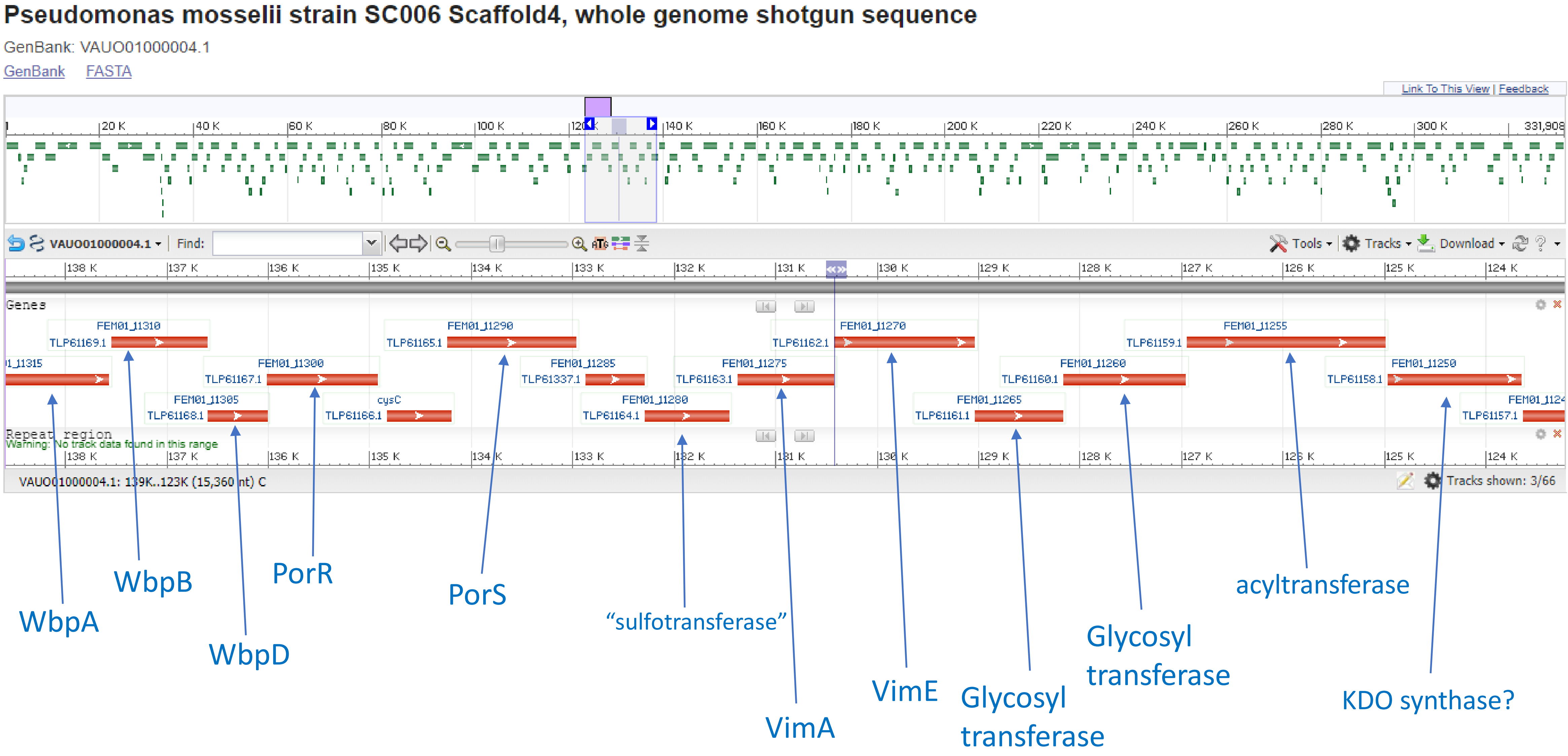

https://www.ncbi.nlm.nih.gov/nuccore/VAUO01000004.1?report=graph&from=130421&to=131380&strand=true

Aeromonas veronii strain FC951 chromosome, complete genome

GenBank: CP032839.1

GenBank FASTA

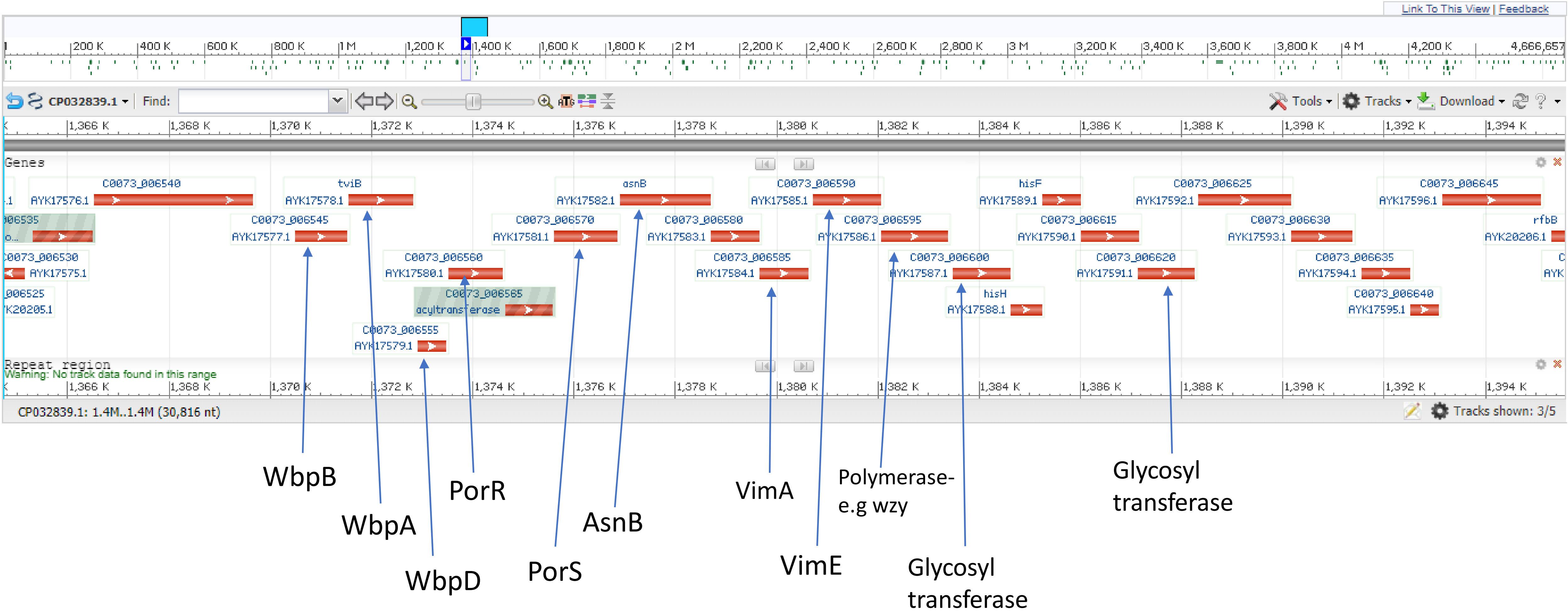

Rhombosortase = C0073_012080

https://www.ncbi.nlm.nih.gov/nuccore/CP032839.1?report=graph&log$=seqview&from=1379665&to=1380627

Vibrio cholerae strain S12 VCS12_contig000003_Vibrio_cholerae_strain_S-12, whole genome shotgun sequence

GenBank: MDST01000003.1

GenBank FASTA

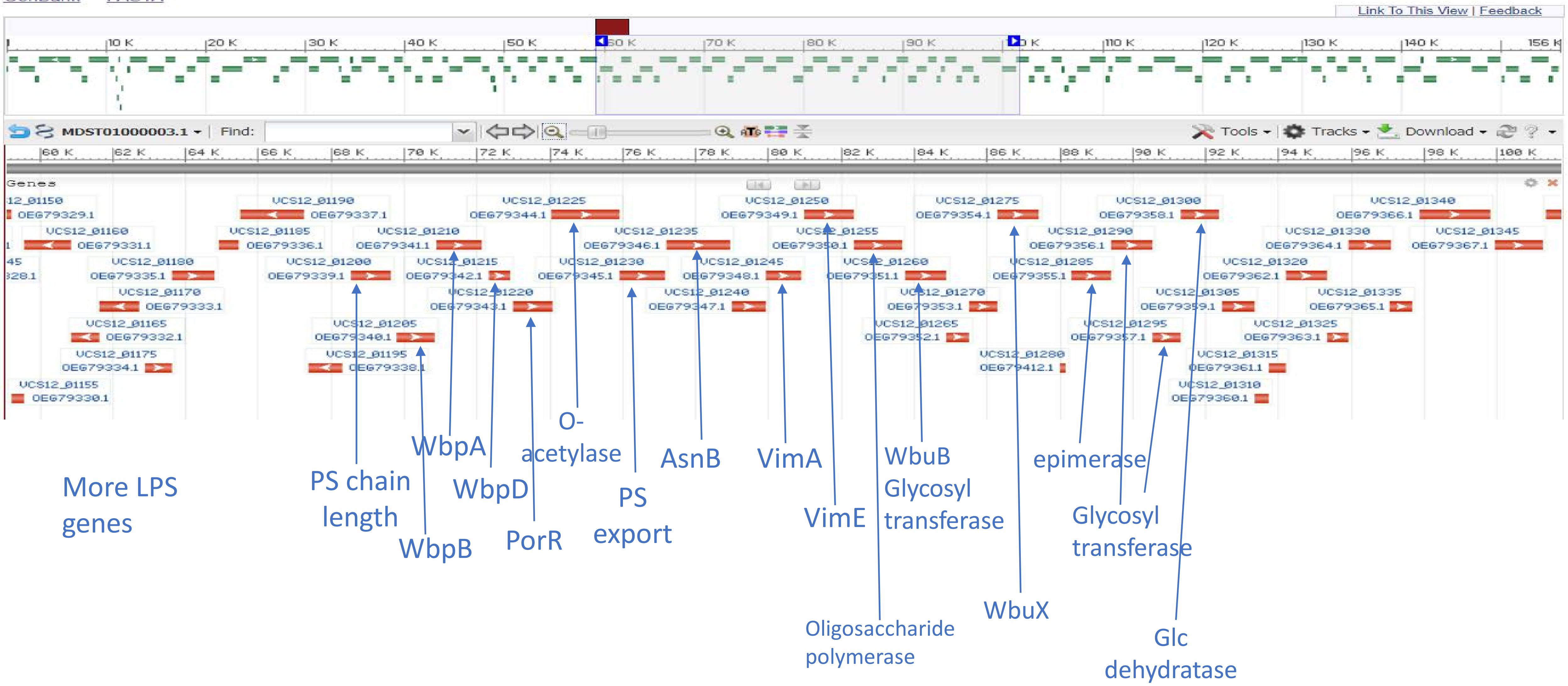

Rhombosortase = VCS12_09380

https://www.ncbi.nlm.nih.gov/nuccore/MDST01000003.1?report=graph&from=79938&to=80900
