# Supplementary material for: Type IX Secretion System Cargo Proteins Are Glycosylated at the C Terminus with a Novel Linking Sugar of the Wbp/Vim Pathway
Source: mBio. 2020 Sep 1;11(5):e01497-20. doi: 10.1128/mBio.01497-20 (PMC7468200; doi:10.1128/mBio.01497-20)
Supplement: TABLE S1 [file mBio.01497-20-st001.docx]

**Table S1: Bacterial strains and plasmids used in this study**

| **Strain or plasmid** | **Description** | **Reference or source** |
| --- | --- | --- |
| ***E. coli* strain** |  |  |
| S17-1 | RP4-2-Tc::Mu *aph*::Tn7 *recA,* Sm^r^ | (1) |
| XL-1 Blue | Host strain for general cloning | Stratagene |
|  |  |  |
| ***P. gingivalis* strain** |  |  |
| ATCC 33277 | Wild type | ATCC |
| KDP202 | ATCC 33277 *vimA*::*tetQ*, Tc^r^ | (2) |
| KDP1101 | ATCC 33277 *PGN_1234*::*ermF*, Em^r^ | This study |
| KDP1102 | ATCC 33277 *PGN_1234*::*ermF*, pTCB-*PGN_1234^+^,* Em^r^ Tc^r^ | This study |
| KDP1103 | ATCC 33277 *vimA*::*tetQ*, *mfa1*_N_ *mfa2*_C_::*ermF*-p-*vimA*_Tf_*^+^*, Tc^r^ Em^r^ | This study |
| KDP1104 | ATCC 33277 *vimA*::*tetQ*, *mfa1*_N_ *mfa2*_C_::*ermF*, Tc^r^ Em^r^ | This study |
|  |  |  |
| ***T. forsythia* strain** |  |  |
| ATCC 43037 | Wild type | ATCC |
|  |  |  |
| ***E. coli* plasmid** |  |  |
| pKD740 | Ap^r^, Δ*hbp35*::*ermF* in pGEM-T Easy | (3) |
| pUC118-*PGN_1234*_up_ | Ap^r^, contains *PGN_1234*_up_ amplicon in pUC118 | This study |
| pUC118-*PGN_1234*_dw_ | Ap^r^, contains *PGN_1234*_dw_ amplicon in pUC118 | This study |
| p*PGN_1234*_up_-*ermF*-*hbp35*_dw_ | Ap^r^, contains *PGN_1234*_up_-*ermF*-*hbp35*_dw_ region in pGEM-T Easy | This study |
| pKD1401 | Ap^r^, *PGN_1234*::*ermF* in pGEM-T Easy | This study |
| pKD954 | Ap^r^, pBluescript II SK(-)-p (pBSSK-p) | (4) |
| pKD955 | Ap^r^, pBSSK-p-*porK^+^-*T | (4) |
| pUC118-*PGN_1234^+^* | Ap^r^, contains *PGN_1234* coding region in pUC118 | This study |
| pBSSK-p-*PGN1234^+^*-T | Ap^r^, contains p-*PGN1234^+^*-T in pBluescript II SK(-) | This study |
| pAL30 | Ap^r^, contains *ermF* in pGEM-T Easy | (5) |
| pUC118-*mfa1*_N_ | Ap^r^, contains *mfa1*_N_ amplicon in pUC118 | This study |
| pUC118-*mfa2*_C_ | Ap^r^, contains *mfa2*_C_ amplicon in pUC118 | This study |
| pUC118-*mfa2*_C_2_ | Ap^r^, pUC118-*mfa2*_C_ with multiple cloning sites XbaI to KpnI of pUC118 removed | This study |
| pUC118-*mfa1*_N_-*mfa2*_C_ | Ap^r^, contains *mfa1*_N_-*mfa2*_C_ in pUC118 | This study |
| p*mfa1*_N_*-ermF-mfa2*_C_ | Ap^r^, contains *mfa1*_N_*-ermF-mfa2*_C_ in pUC118 | This study |
| pUC118-p-*vimA*_Tf_*^+^* | Ap^r^, contains p-*vimA*_Tf_*^+^* amplicon in pUC118 | This study |
| p*vimA*_Tf_*^+^* | Ap^r^, contains *mfa1*_N_*-ermF-*p-*vimA*_Tf_*^+^-mfa2*_C_ in pUC118 | This study |
|  |  |  |
| ***P. gingivalis* plasmid** |  |  |
| pTCB | Ap^r^, Tc^r^, *E. coli*-*P. gingivalis* shuttle plasmid | (6) |
| pTCB-*PGN1234^+^* | Tc^r^, p-*PGN_1234^+^*-T in KpnI-NotI sites of pTCB | This study |

**References**

1. Simon R, Priefer U, Pühler A. 1983. A broad host range mobilization system for *in vivo* genetic engineering: Transposon mutagenesis in gram negative bacteria. Biotechnology (N Y) 1:784-791.

2. Shoji M, Sato K, Yukitake H, Kondo Y, Narita Y, Kadowaki T, Naito M, Nakayama K. 2011. Por secretion system-dependent secretion and glycosylation of *Porphyromonas gingivalis* hemin-binding protein 35. PLoS One 6:e21372.

3. Shoji M, Shibata Y, Shiroza T, Yukitake H, Peng B, Chen YY, Sato K, Naito M, Abiko Y, Reynolds EC, Nakayama K. 2010. Characterization of hemin-binding protein 35 (HBP35) in *Porphyromonas gingivalis*: its cellular distribution, thioredoxin activity and role in heme utilization. BMC Microbiol 10:152.

4. Sato K, Naito M, Yukitake H, Hirakawa H, Shoji M, McBride MJ, Rhodes RG, Nakayama K. 2010. A protein secretion system linked to bacteroidete gliding motility and pathogenesis. Proc Natl Acad Sci U S A 107:276-81.

5. Dashper SG, Ang CS, Veith PD, Mitchell HL, Lo AW, Seers CA, Walsh KA, Slakeski N, Chen D, Lissel JP, Butler CA, O'Brien-Simpson NM, Barr IG, Reynolds EC. 2009. Response of *Porphyromonas gingivalis* to heme limitation in continuous culture. J Bacteriol 191:1044-55.

6. Nagano K, Murakami Y, Nishikawa K, Sakakibara J, Shimozato K, Yoshimura F. 2007. Characterization of RagA and RagB in *Porphyromonas gingivalis*: study using gene-deletion mutants. J Med Microbiol 56:1536-1548.
