# Supplementary material for: Type IX Secretion System Cargo Proteins Are Glycosylated at the C Terminus with a Novel Linking Sugar of the Wbp/Vim Pathway
Source: mBio. 2020 Sep 1;11(5):e01497-20. doi: 10.1128/mBio.01497-20 (PMC7468200; doi:10.1128/mBio.01497-20)
Supplement: TABLE S2 [file mBio.01497-20-st002.pdf]

Table S2: Primers used in this study.

Name	Sequence[a]
PGN1234upFw	GCATGCTGGCCGAACAGAAGAATACCCGAC (Underline indicates SphI site)
PGN1234upRev	GGATCCGGCACGTTGGAATACGACAGCCCA (Underline indicates BamHI site)
PGN1234dwFw	CTGCAGTGTATATCAGGCCAAGCAAAGCCA (Underline indicates PstI site)
PGN1234dwRev	GAGCTCGTTTCCTGAGAGCTTCTCACTTGG (Underline indicates SacI site)
PGN1234compFw	GTCGACATGAAAATAGTCTCAGACTTCACT (Underline indicates SalI site)
PGN1234compRev	TCTAGATTAGAGAGGCAGAGGGCGGTCAAT (Underline indicates XbaI site)
mfa1-Fw	GCATGCTCTCATTGGGCTTTGCTTCT (Underline indicates SphI site)
mfa1-R	GGATCCGCATCAAAGTCTGCTGCATT (Underline indicates BamHI site)
mfa2-F	GGATCCCTGCAGATGGCGCGGATCAGTATA (Underline indicates BamHI-PstI sites)
mfa2-R	GAGCTCATCAACCAGCCGTTTATCCA (Underline indicates SacI site)
Pcat-F	GGTACCTTCGTCGTCAATCAGCATCCCAG (Underline indicates KpnI site)
Pcat-R	CGGGACATTGTTTTGTCTCTTATTTAAGTTA
TfvimA-F	ACAAAACAATGTCCCGCAAAGAAACTTATCGTACAT
TfvimA-R	GCGGCCGCTCATCTCGTTTGCGCCCATTTG (Underline indicates NotI site)

[a]Underlined sequence represents non-homologous sequence for introduction of restriction sites.
