# Supplementary material for: Type IX Secretion System Cargo Proteins Are Glycosylated at the C Terminus with a Novel Linking Sugar of the Wbp/Vim Pathway
Source: mBio. 2020 Sep 1;11(5):e01497-20. doi: 10.1128/mBio.01497-20 (PMC7468200; doi:10.1128/mBio.01497-20)
Supplement: TABLE S3 [file mBio.01497-20-st003.pdf]

Table S3. PGN_1234 PSI-BLAST matches to WbpS and WbqG compared to AsnB

Species	[a]NCBI	[b]Uniprot	Name	[c]E-value	[d]% cov, % id	[e]CD top hit
P. aeruginosa	WP_012613869.1	Q9RHD2_PSEAI	WbpS	$6E^{-79}$	92, 17.3	eps_aminotran_1
E. coli	WP_032178609.1	J3RWI0_ECOLX	WbqG	$6E^{-76}$	97, 17.2	eps_aminotran_1
P. aeruginosa	WP_003115546.1	Q9I231_PSEAE	AsnB	$3E^{-61}$	88, 14.3	AsnB
E. coli	WP_000337077.1	ASNB_ECOLI	AsnB	$1E^{-43}$	92, 15.1	AsnB

BLAST settings: PSI-BLAST, 2 iterations; taxonomy = gamma proteobacteria; max # hits = 10,000; Database = NCBI nr; Input sequence = PGN_1234.

[a]The NCBI accessions are non-redundant sequences that match to the same protein from many different strains.

[b]The uniprot accessions are unique and match the protein name provided

[c]E-value = Expect Value

[d]% cov = coverage of sequence (%); % id = level of sequence identity (%)

[e]The top hit to the conserved domain database (https://www.ncbi.nlm.nih.gov/Structure/cdd/wrpsb.cgi)
